# ZFPL1 Promotes Colorectal Cancer Progression by Stabilizing ASS1 to Drive the Urea Cycle and M2 Macrophage‐Mediated Metastatic Colonization

**DOI:** 10.1002/advs.202505291

**Published:** 2025-11-11

**Authors:** Xiangjun Qian, Chenxi Xie, Beilin Zhang, Hengsong Cao, Zhengqing Lu, Li Liu, Shipeng Dai, Xiaoqian Wang, Xiaokai Zhang, Feng Han, Yanyan Liu, Haibo Yu, Weiwei Tang, Jinxue Zhou, Xiaopei Hao

**Affiliations:** ^1^ Department of Hepatobiliary and Pancreatic Surgery The Affiliated Cancer Hospital of Zhengzhou University & Henan Cancer Hospital 7 Weiwu Road zhengzhou 450000 China; ^2^ Department of Hepatobiliary and Pancreatic Surgery People's Hospital of Zhengzhou University Zhengzhou China; ^3^ Hepatobiliary Center Key Laboratory of Liver Transplantation Chinese Academy of Medical Sciences NHC Key Laboratory of Hepatobiliary cancers The First Affiliated Hospital of Nanjing Medical University Nanjing China; ^4^ First Teaching Hospital of Tianjin University of Traditional Chinese Medicine Tianjin China; ^5^ National Clinical Research Center for Chinese Medicine Acupuncture and Moxibustion Tianjin China

**Keywords:** ASS1, colorectal cancer, liver metastases, single‐cell RNA sequencing, tumor microenvironment, urea metabolism, ZFPL1

## Abstract

Colorectal cancer (CRC) progression is driven by diverse molecular mechanisms, underscoring the urgent need for novel therapeutic strategies, especially for liver metastases. Through an integrated analysis of multiple single‐cell RNA sequencing databases, zinc finger protein‐like 1 (ZFPL1) is identified as a gene specifically enriched in malignant cells from both primary and metastatic CRC. Multi‐omics investigations demonstrate that high ZFPL1 expression correlates with aggressive clinicopathological features and poor survival. Functionally, ZFPL1 promotes tumor proliferation, invasion, and migration both in vivo and in vitro. Mechanistically, ZFPL1 directly binds argininosuccinate synthase 1 (ASS1), shielding its K57 residue from tripartite motif containing 33‐mediated ubiquitination to prevent proteasomal degradation. This stabilization activates urea cycle metabolism, driving CRC progression. Crucially, ZFPL1 deficiency remodels the tumor microenvironment by reducing immunosuppressive populations‐M2 macrophages, and promoting pro‐inflammatory M1 polarization. Virtual screening identifies Salvianolic acid B (Sal B) as a ZFPL1 inhibitor, which disrupts ZFPL1‐ASS1 binding, triggering ASS1 ubiquitination and degradation. In vivo, Sal B synergized with anti‐PD‐1 therapy, significantly reducing tumor burden versus monotherapy. These findings establish ZFPL1 as a key regulator of CRC progression through ASS1‐dependent urea cycle activation and immunomodulation, nominating the ZFPL1‐ASS1 axis as a therapeutic target, with Sal B demonstrating combinatorial potential with immunotherapy.

## Graphical abstract

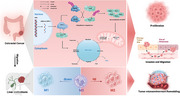

## 1 Introduction

Colorectal cancer (CRC) represents a significant global health burden, ranking as the third most commonly diagnosed malignancy and the second leading cause of cancer‐related mortality worldwide. Epidemiological data from 2022 indicate a substantial disease burden, with over 1.9 million new cases and 904 000 deaths reported.^[^ ^1^ ^]^ Notably, recent epidemiological trends have revealed a concerning increase in CRC incidence among younger populations, particularly those under 50 years of age. Projections suggest a dramatic rise in incidence rates for colon and rectal cancers among individuals aged 20–34 years, with anticipated increases of 90.0% and 124.2%, respectively.^[^ ^2^, ^3^ ^]^ The pathogenesis of CRC has been extensively studied, with multiple well‐established risk factors identified. These include inflammatory bowel disease, tobacco use, dietary factors (particularly high consumption of red and processed meats), excessive alcohol intake, diabetes mellitus, sedentary lifestyle, metabolic syndrome, and obesity (as measured by elevated body mass index).^[^ ^1^, ^4^ ^]^ Current therapeutic approaches for CRC are stratified according to disease stage and characteristics. For localized disease, encompassing malignant colorectal polyps and resectable non‐metastatic CRC, primary interventions include endoscopic polypectomy and surgical resection. In cases demonstrating advanced disease characteristics or high risk of systemic recurrence, adjuvant or neoadjuvant chemotherapy regimens are typically incorporated into the treatment paradigm.^[^ ^5^ ^]^ Despite advances in early detection and treatment, metastatic progression remains a major clinical challenge in CRC management. Epidemiological studies indicate that 50–60% of CRC patients develop metastatic disease, with ≈80% of these cases presenting with unresectable hepatic metastases.^[^ ^6^, ^7^ ^]^ The liver represents the most frequent site of metastatic involvement, with metastases often developing metachronously following treatment of the primary tumor.^[^ ^8^ ^]^ Postmortem analyses reveal that more than 50% of CRC‐related deaths are associated with hepatic metastases. The prognostic implications of metastatic CRC are particularly severe, with non‐surgical candidates demonstrating markedly reduced 5‐year survival rates compared to those amenable to surgical intervention.^[^ ^6^, ^9^ ^]^ These clinical realities underscore the urgent need for continued development of novel therapeutic strategies in CRC management, particularly for liver metastatic disease, which remains a critical area of investigation in contemporary oncological research.

Liver metastasis remains a major obstacle to the long‐term survival of patients with CRC, which can be partially attributed to the highly dynamic dissemination pathways of cancer cells. Beyond the cancer cells themselves, the tumor microenvironment (TME) of liver metastases exhibits a highly immunosuppressive phenotype, leading to systemic depletion of antigen‐specific T lymphocytes and facilitating tumor dissemination.^[^ ^10^ ^]^ Tumor‐associated macrophages (TAMs) comprehensively influence the cellular composition and metabolic characteristics of the TME by producing immunosuppressive cytokines (such as IL‐10 and TGF‐β) that inhibit CD8⁺ T cell function and by recruiting immunomodulatory cell populations. However, the underlying mechanisms of this intricate crosstalk among tumor cells, stromal cells and TAMs remain incompletely understood.^[^ ^11^ ^]^ Although studies have shown that TAMs are associated with poor prognosis in most solid tumors, they may exert context‐dependent pro‐metastatic or anti‐metastatic roles in CRC. Emerging evidence indicates a correlation between TAM density and colorectal cancer liver metastasis, confirming TAMs as a key determinant of immune tolerance in liver metastases. For example, SPHK1^+^ TAMs induce CD8^+^ T cell dysfunction and immune evasion in CRC.^[^ ^12^ ^]^ Paradoxically, a microarray analysis of 159 specimens from Crohn's‐like colorectal cancer patients revealed that higher expression levels of CD68^+^ TAMs were associated with improved prognosis.^[^ ^13^ ^]^ These conflicting findings highlight the heterogeneity in the polarization of TAMs, which is modulated by signals such as cytokines and metabolites within the tumor microenvironment. Furthermore, the dynamic trajectory of TAMs within this complex microenvironment and the key upstream drivers of their behavior remain largely uncharacterized.^[^ ^14^ ^]^ Therefore, investigating which factors in the TME influence the polarization of TAMs and elucidating how they affect this polarization are crucial for developing novel therapeutic strategies aimed at reversing macrophage polarization and suppressing CRC growth.

The advent of single‐cell RNA sequencing (scRNA‐seq) technology has revolutionized our understanding of genetics and tumor biology through its unparalleled capacity to delineate the epigenomic, transcriptomic, and genomic landscapes at single‐cell resolution. This technological breakthrough has enabled researchers to systematically investigate the dynamic molecular alterations occurring throughout tumor progression, including the complex biological processes underlying advanced metastatic cancers.^[^ ^15^, ^16^ ^]^ From a clinical perspective, the implementation of scRNA‐seq technology holds transformative potential for cancer treatment paradigms. Particularly noteworthy is its significant contribution to the comprehensive characterization of the tumor microenvironment (TME), coupled with the identification of novel therapeutic targets.^[^ ^17^ ^]^ In the present study, our analysis of scRNA‐seq data revealed a distinct enrichment of zinc finger protein‐like 1 (ZFPL1) in malignant CRC cells compared to other cellular populations. Mechanistically, ZFPL1 was found to inhibit ubiquitin‐mediated degradation of asparagine synthetase 1 (ASS1) at lysine 57 (K57), resulting in ASS1 upregulation and subsequent activation of urea cycle metabolism. Mass cytometry analysis demonstrated that the deficiency of ZFPL1 leads to the depletion of M2 macrophages and promotes M1 polarization in the TME of CRC. Through computational drug screening, we identified Salvianolic acid B (Sal B) as a potent ZFPL1 inhibitor, and subsequent experimental validation confirmed its ability to disrupt ZFPL1‐ASS1 interaction and suppress CRC malignancy. Furthermore, Sal B synergized effectively with anti‐PD‐1 immunotherapy in vivo, highlighting the therapeutic potential of targeting the ZFPL1‐ASS1 pathway in combination with immune checkpoint blockade for advanced CRC.

## 2 Results

### 2.1 ScRNA‐seq Reveals ZFPL1 as a Tumor Cell‐Restricted Gene in CRC Linked to Poor Survival

**Figure 1:**
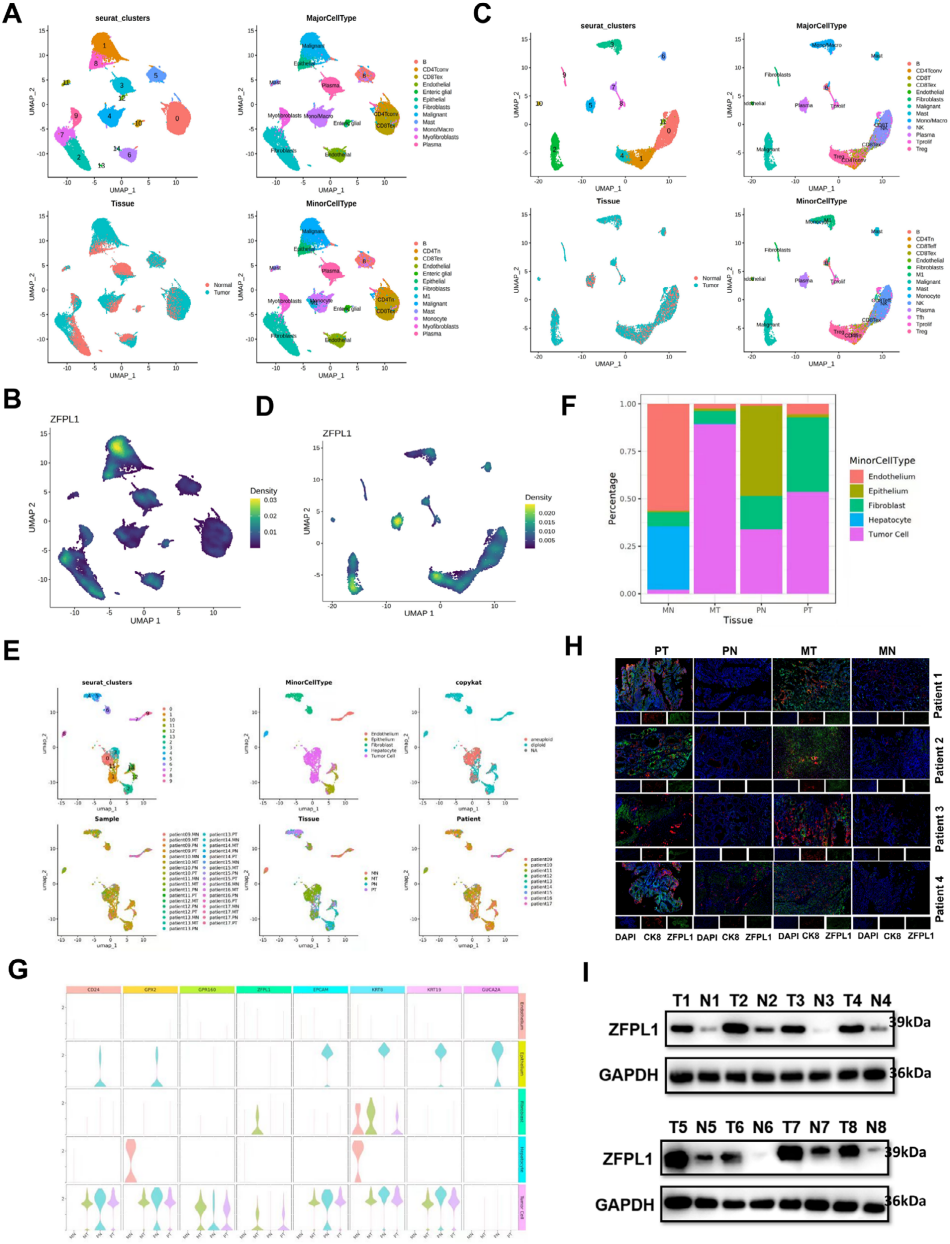
ScRNA‐seq Reveals ZFPL1 as a Tumor Cell‐Restricted Gene in CRC Linked to Poor Survival. A) Based on the classification of specific gene markers using UMAP plots, EMTAB8107 results reveal 12 cell clusters in CRC. B) The UMAP plot showing ZFPL1 was highly enriched in malignant cells of CRC based on EMTAB8107 data. C) Based on UMAP plots, GSE146771 results reveal 13 cell clusters in CRC. D) The UMAP plot showing ZFPL1 was highly enriched in malignant cells of CRC based on GSE146771. E) Using UMAP and dot plots, specific gene markers were classified as defined in GSE164522. Results indicating that a total of 5 cell clusters in CRC with liver metastasis. F) The bar chart depicts the expression levels of different cell types in the primary CRC lesion (PT), the secondary liver lesion (MT), and the respective adjacent normal tissues(PN, MN) using data from GSE164522. G) A violin plot generated using the GSE164522 dataset is presented in Figure 1E, illustrating the distribution of genes with specific expression patterns across various cell clusters. H) Pathologists with professional expertise evaluated immunohistochemistry images of the puncture samples from two primary CRC patients to determine if the samples contained tumor tissue. Statistical analysis of the images of each group was conducted using ImageJ. I) Western blot analysis was conducted to assess ZFPL1 protein levels in eight pairs of CRC tissues (T) and their adjacent non‐cancerous tissues(N). GAPDH is used for loading control. *n* = 8 independent biological replicates.

Through comprehensive scRNA‐seq analysis, we identified ZFPL1 as a specifically enriched gene in CRC tumor cells. Our investigation encompassed three independent scRNA‐seq datasets, systematically characterizing ZFPL1 expression patterns across different cellular compartments in CRC. Initial analysis of the EMTAB8107 dataset revealed 12 distinct cell clusters, including B cells, T cell subsets (CD4^+^ and CD8^+^), fibroblasts, malignant cells, and epithelial cells. Through UMAP visualization and specific gene marker identification (**Figure** **1**A; Figure S1A,B, Supporting Information), we observed significant ZFPL1 enrichment specifically in malignant cell populations, with minimal expression in other cellular subsets (Figure 1B). This finding was further substantiated by analysis of the GSE146771 dataset, which identified 13 cellular clusters, including additional immune cell populations such as T regulatory cells (Tregs) and mast cells. Consistent with our initial observations, ZFPL1 maintained its specific expression pattern in malignant cells (Figure 1C,D; Figure S1C,D, Supporting Information). To explore ZFPL1 expression in metastatic CRC, we analyzed the GSE164522 dataset, which included samples from both primary CRC tumors (PT) and their corresponding liver metastases (MT). Notably, ZFPL1 demonstrated consistent enrichment in tumor cells from both primary and metastatic sites(PN, MN), while showing negligible expression in adjacent normal tissues (Figure 1E–G; Figure S1E, Supporting Information). Furthermore, we analyzed five samples obtained from one CRC patient with liver metastasis, including the primary tumor, matched adjacent normal tissue, liver metastasis, adjacent normal tissue to the metastasis, and blood using scRNA‐seq. UMAP clustering identified five major cell populations: endothelial cells, epithelial cells, fibroblasts, lymphocytes, and myeloid cells (Figure S2A,B, Supporting Information). Subsequent subclustering analysis of the myeloid cells and stromal cells revealed moderate enrichment of ZFPL1 within the tumor cell clusters (Figure S2C–I, Supporting Information).

To validate these scRNA‐seq findings at the protein level, we performed immunofluorescence on biopsy samples from 4 primary CRC patients with liver metastases, comparing tumor tissues with adjacent non‐tumoral counterparts. This orthogonal validation approach revealed significantly higher ZFPL1 expression in tumor cells (CK8 positive) compared to adjacent tissues (Figure 1H). Western blot analysis of 8 paired CRC and normal tissue samples consistently demonstrated elevated ZFPL1 protein levels in tumor tissues (Figure 1I). Furthermore, immunohistochemical staining of tumor tissues from 80 CRC patients demonstrated that high ZFPL1 expression was significantly associated with clinical lymph node metastasis, lymphovascular invasion, and increased tumor diameter, while showing no correlation with other factors such as TNM stage or KRAS mutation status (Table S1, Supporting Information). Prognostic analysis indicated that high ZFPL1 expression was significantly associated with poor disease‐free survival (DFS) and overall survival (OS) (Figure S3A–C, Supporting Information). Analysis of public databases further corroborated these findings. Although no significant difference in OS was observed in these external cohorts (Figure S3D, Supporting Information), they showed that CRC patients with high ZFPL1 expression had reduced relapse‐free survival (RFS) (Figure S3E, Supporting Information). Together, our integrated multi‐omics analysis establishes ZFPL1 as a tumor cell‐enriched gene in CRC, validated across scRNA‐seq datasets and protein‐level assays, whose high expression correlates with aggressive clinicopathological features and poor patient survival outcomes.

### 2.2 ZFPL1 Drives CRC Malignancy In Vitro and In Vivo

**Figure 2:**
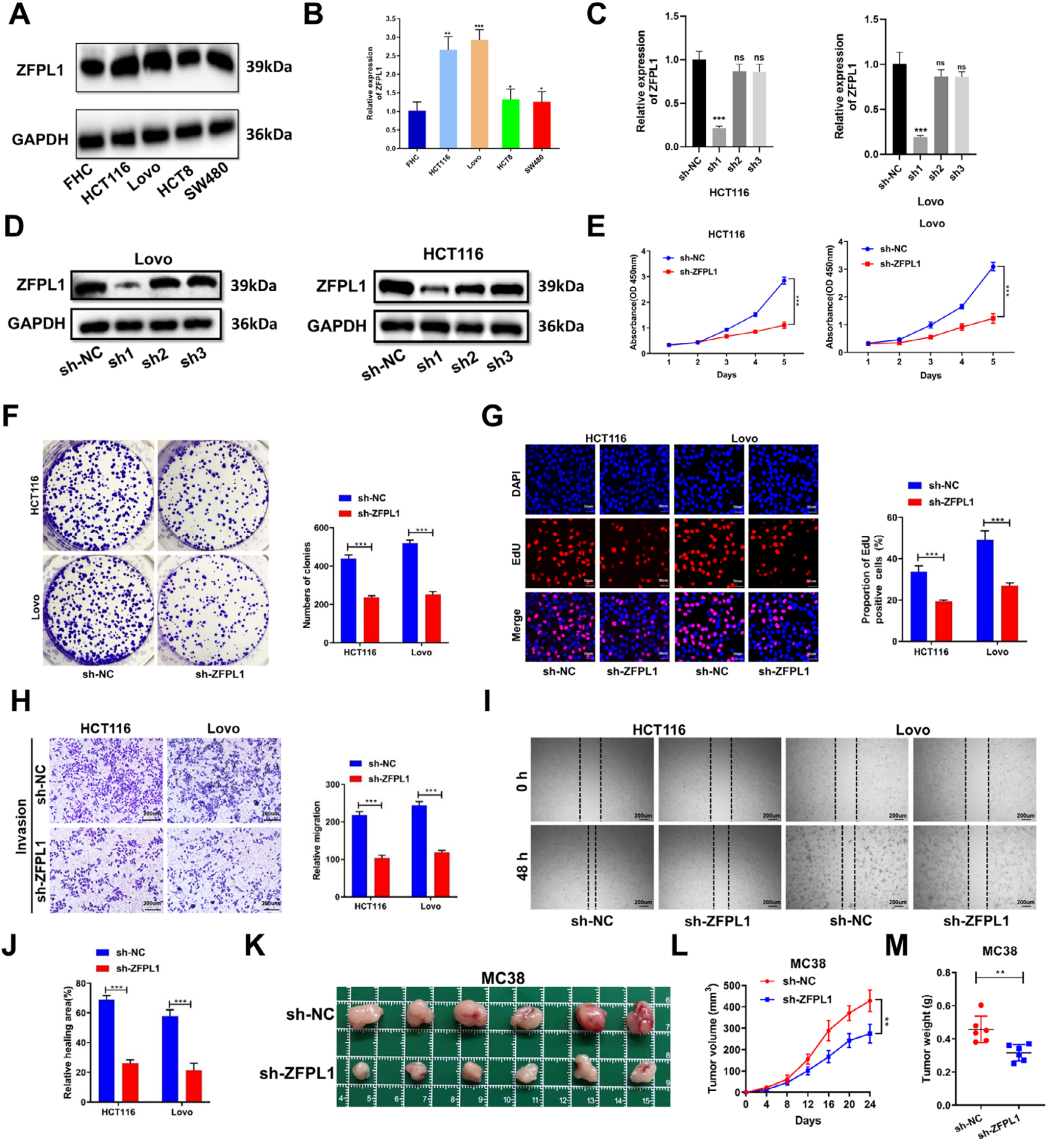
ZFPL1 Drives CRC Malignancy In Vitro and In Vivo. A) Using western blotting to evaluate the protein expression level of ZFPL1 in CRC cell lines. GAPDH is used for loading control. *n* = 3 independent biological replicates. B) Using qRT‐PCR to assess the mRNA expression level of ZFPL1 in CRC cell lines. *n* = 3 independent biological replicates. C) Assessing the knockdown effect of shRNA transfection on the HCT116 and Lovo by qRT‐PCR. *n* = 3 independent biological replicates. D) Assessing the knockdown effect of shRNA transfection on the HCT116 and Lovo by western blot. GAPDH is used for loading control. *n* = 3 independent biological replicates. E) CCK‐8 assays were conducted to evaluate ZFPL1's impact on CRC cell proliferation. *n* = 3 independent biological replicates. F) Colony formation assay assessed the impact of ZFPL1 on CRC cell proliferation. *n* = 3 independent biological replicates. G) EdU assays were conducted to evaluate the impact of ZFPL1 on the proliferation of CRC cells (Scale bar, 50 µm). *n* = 3 independent biological replicates. H) To evaluate the migration and invasion capabilities of transfected CRC cell lines, Transwell assays were conducted (Scale bar, 200 µm). *n* = 3 independent biological replicates. I,J) Wound healing assays were conducted to assess the impact of ZFPL1 knockdown on CRC cell migration (Scale bar, 200 µm). *n* = 3 independent biological replicates. K) Representative images of subcutaneous xenograft tumors. *n* = 6 independent biological replicates. The weight and volume of subcutaneous xenograft tumors demonstrate the impact of ZFPL1 knockdown on CRC formation. L) Growth curves of xenograft tumor volumes. *n* = 6mice per group. M) Graphs of xenograft tumor weights. *n* = 6 mice per group. In all statistical plots, data are shown as mean ± SEM, one‐way ANOVA (Figure 2B,C,E,L) and Student's t test (Figure 2F,G,J,K,M) were used to determine statistical significance (ns = not significant, *p < 0.05; **p < 0.01; ***p < 0.001).

To investigate ZFPL1's role in CRC, we first assessed its expression across multiple CRC cell lines via western blot and qRT‐PCR. Comparative analysis showed significantly elevated ZFPL1 expression in HCT116 and Lovo cells relative to normal colorectal mucosal cells (FHC), while HCT8 cells exhibited a lower expression (**Figure** **2**A,B). Based on this expression pattern, we selected these three cell lines for subsequent functional studies. To elucidate the functional impact of ZFPL1 on CRC cells, we developed three shRNA sequences targeting ZFPL1 (sh‐ZFPL1) in HCT116 and Lovo cell lines and a lentiviral vector of ZFPL1 in HCT8 cells. Among the three shRNA sequences designed, sh1 demonstrated the most efficient knockdown of both ZFPL1 mRNA and protein levels (Figure 2C,D), and was therefore selected to establish stable ZFPL1‐knockdown cell lines for further experiments. The lentiviral vector significantly upregulated the expression of ZFPL1 (Lv‐ZFPL1) (Figure S4A,B, Supporting Information). Functional assays revealed that ZFPL1 knockdown significantly impaired CRC cell proliferation, as evidenced by CCK‐8 (Figure 2E), colony formation (Figure 2F), and EdU incorporation assays (Figure 2G). Furthermore, transwell and wound healing assays demonstrated that ZFPL1 suppression markedly reduced the invasive and migratory capacities of CRC cells (Figure 2H–J). Conversely, overexpression of ZFPL1 in HCT8 cells led to enhanced proliferation (Figure S4C–E, Supporting Information), invasion, and migration (Figure S4F,G, Supporting Information), further supporting the oncogenic role of ZFPL1 in CRC progression. To further explore the role of ZFPL1 in vivo, we established a subcutaneous tumor model by injecting MC38 cells with ZFPL1 knockdown (sh‐ZFPL1) or control (sh‐NC) into C57BL/6 mice. The sh‐ZFPL1 group exhibited significantly reduced tumor volume and weight compared to the sh‐NC group (Figure 2K–M). Additionally, in a CRC liver metastasis model, ZFPL1 knockdown markedly attenuated the metastatic potential of CRC cells (Figure S4H, Supporting Information). Collectively, these results demonstrate that ZFPL1 knockdown effectively suppresses CRC progression both in vitro and in vivo, highlighting its potential as a therapeutic target.

### 2.3 ZFPL1 Drives CRC Progression via ASS1‐Dependent Urea Cycle Activation

**Figure 3:**
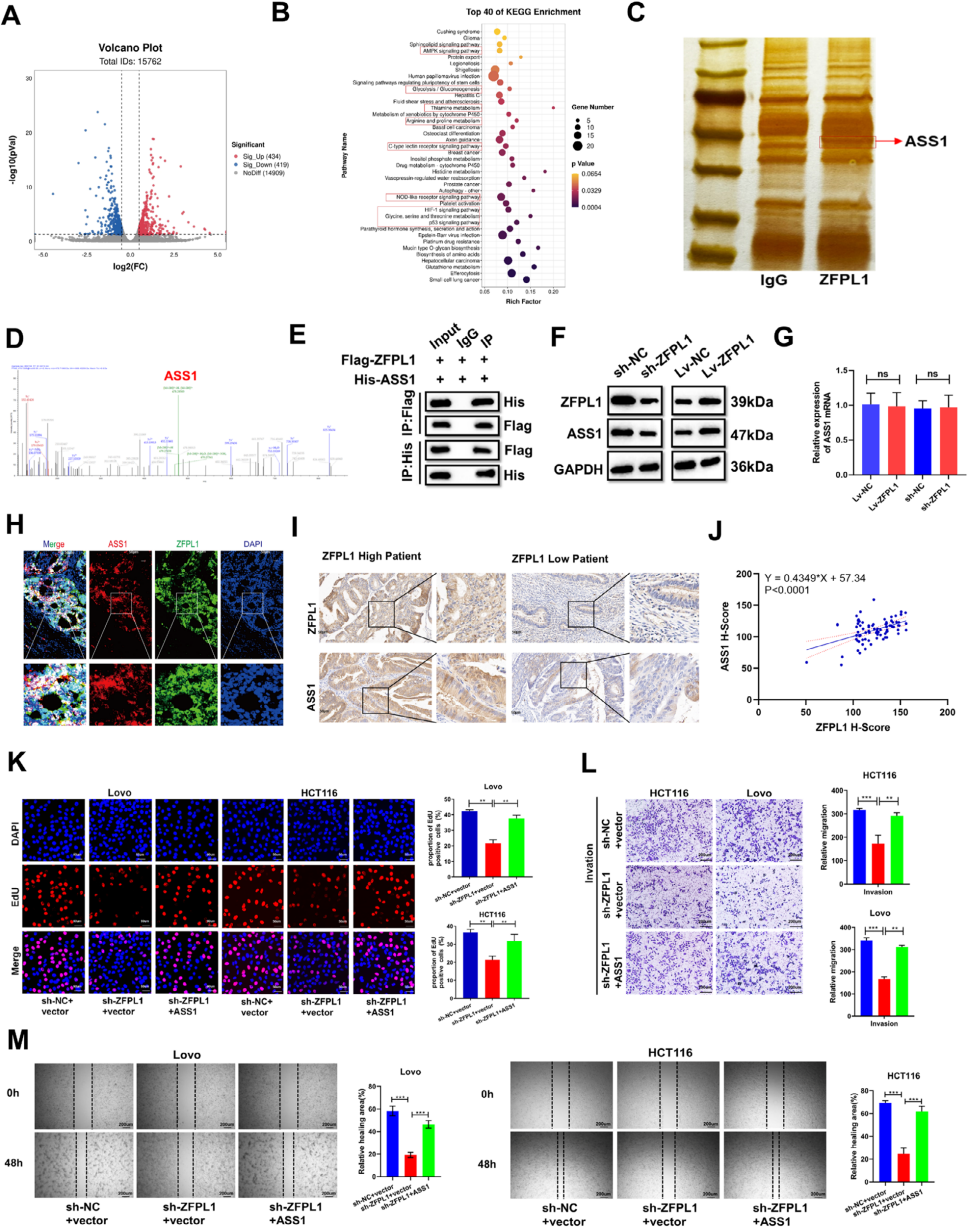
ZFPL1 Drives CRC Progression via ASS1‐Dependent Urea Cycle Activation. A) Volcano plot was used to illustrate the genes with differential expression following the knockdown of ZFPL1 in CRC cells. B) KEGG analysis revealed that downregulated genes were predominantly enriched in arginine and proline metabolism, HIF‐1, and cancer pathways. C) Proteins from SDS‐PAGE gels derived from IP products of ZFPL1 and negative control were extracted for mass spectrometry analysis. The red rectangle serves as the target area for mass spectrometry analysis. *n* = 3 independent biological replicates. D) Typical peptides were identified in the ZFPL1‐enriched proteins through mass spectrometry analysis. E) Co‐IP analysis was performed to demonstrate the interaction between ZFPL1 and ASS1 in HEK293T cells transfected with His‐tagged ASS1 and Flag‐tagged ZFPL1. *n* = 3 independent biological replicates. F) Western blot analysis evaluated ASS1 expression in CRC cell lysates with ZFPL1 knockdown and overexpression. GAPDH served as the loading control. *n* = 3 independent biological replicates. G) qRT‐PCR analysis was conducted to assess the expression of ASS1 in lysates prepared from CRC cells with ZFPL1 knockdown and overexpression. *n* = 3 independent biological replicates. H) Fluorescent immunohistochemical images demonstrate the localization of ZFPL1 and ASS1 in CRC tissue. (Scale bar, 50 µm). *n* = 80 independent biological replicates. I) The immunohistochemical analysis revealed a significant difference in the expression pattern of ASS1 between the high and low expression groups of ZFPL1 in clinical samples of CRC. (Scale bar, 50 µm). *n* = 80 independent biological replicates. J) Pearson correlation assay analyzed the correlation between the expression of ZFPL1 and ASS1 (*n* = 80). K) EdU assays were carried out to assess how ZFPL1's regulation of ASS1 affects the proliferation of CRC cells (Scale bar, 50 µm). *n* = 3 independent biological replicates. L) Transwell assays were implemented to gauge the influence of ZFPL1's regulation of ASS1 on the migration and invasion capabilities of transfected CRC cell lines (Scale bar, 200 µm). *n* = 3 independent biological replicates. M) Wound healing assays were performed to evaluate the effect of ZFPL1 on ASS1 regulation, particularly with respect to the migration and invasion abilities of the transfected CRC cell lines (Scale bar, 200 µm). *n* = 3 independent biological replicates. In all statistical plots, data are shown as mean ± SEM, Student's t test (Figure 3G,K–M) were used to determine statistical significance (ns = not significant, **p < 0.01; ***p < 0.001).

To elucidate the molecular mechanisms underlying ZFPL1‐induced CRC progression, we conducted integrated transcriptomic analyses to determine the downstream genes influenced by ZFPL1. RNA sequencing revealed significant alterations in gene expression profiles following ZFPL1 knockdown, as demonstrated by the volcano plot (**Figure** **3**A). Subsequent KEGG pathway analysis identified significant enrichment of urea cycle metabolism, glucose metabolism, and cancer‐related pathways (Figure 3B), suggesting potential involvement of these pathways in ZFPL1‐mediated CRC progression. Next, we performed an Immunoprecipitation (IP) assay and mass spectrometry analysis to identify potential ZFPL1‐interacting proteins in CRC cells. IP coupled with mass spectrometry identified ASS1 (a rate‐limiting enzyme in arginine biosynthesis) as a potential interacting protein of ZFPL1, which was subsequently validated through Co‐immunoprecipitation (Co‐IP) experiments (Figure 3C–E). To investigate the correlation between ZFPL1 and ASS1 expression, qRT‐PCR and western blot were performed and demonstrated a positive correlation between ZFPL1 and ASS1 protein expression, independent of ASS1 mRNA levels, in both ZFPL1‐knockdown and overexpressing cell lines (Figure 3F,G). Furthermore, immunofluorescence demonstrated ZFPL1‐ASS1 co‐localization in human CRC tissues (Figure 3H). Subsequently, we conducted immunohistochemical analysis on clinical CRC samples and observed a positive correlation between ZFPL1 and ASS1 expression, which is consistent with the western blot findings (Figure 3I,J).

The expression profile of ASS1 demonstrates remarkable heterogeneity across various cancer types, which fundamentally dictates their arginine auxotrophic phenotypes. Comprehensive analysis reveals that complete loss of ASS1 expression, observed in multiple malignancies including melanoma, hepatocellular carcinoma (HCC), renal cell carcinoma, glioblastoma, bladder cancers, and prostate cancer, results in absolute dependence on exogenous arginine supply.^[^ ^18^ ^]^ Conversely, specific cancer types such as CRC, ovarian cancer, and gastric cancer exhibit partial arginine auxotrophy, as demonstrated by their residual ASS1 enzymatic activity and maintained endogenous arginine biosynthesis capability.^[^ ^19^, ^20^ ^]^ The above studies suggest that the urea cycle activation caused by upregulation of ASS1 expression plays an important promoting role in the progression of CRC. To investigate whether the promoting effect of ZFPL1 on CRC is mediated through upregulation of ASS1 expression, we conducted rescue experiments through ASS1 co‐transfection in ZFPL1‐knockdown cells. The results demonstrated that ASS1 overexpression effectively restored cellular proliferation (Figure 3K), invasion, and migration capacities (Figure 3L,M) in ZFPL1‐deficient cells, suggesting that the promoting effect of ZFPL1 on CRC progression is significantly dependent on the upregulation of ASS1 protein levels.

**Figure 4:**
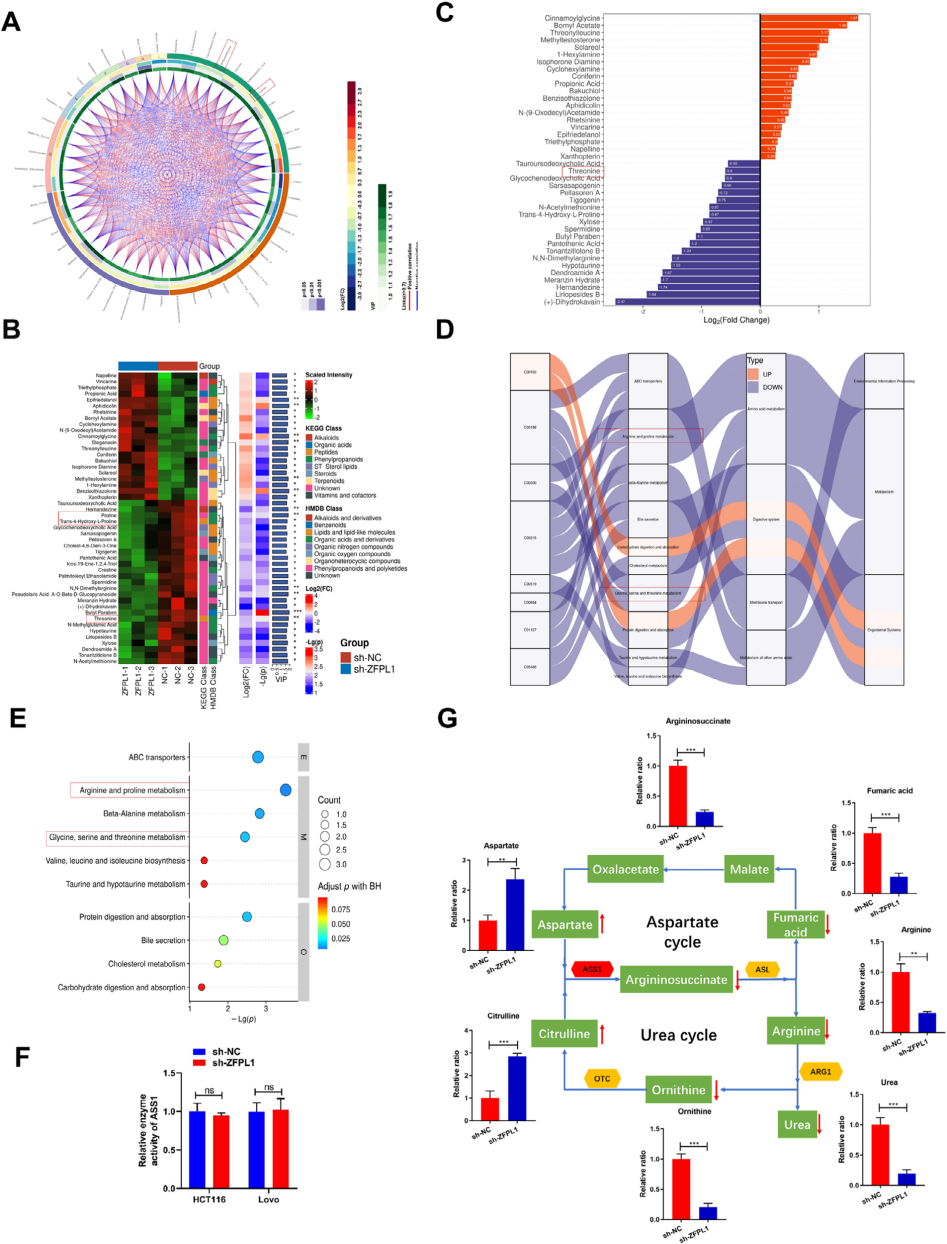
ZFPL1 Activates the Urea Cycle to Promote the Progression of CRC. A) The cycle diagram primarily illustrates the relationships among various differential metabolites. B) The metabolites in each comparison group were classified and quantified based on their structural and functional attributes, with the classification results sourced from the KEGG and HMDB databases. C) The most significantly up‐regulated metabolites following ZFPL1 knockdown in CRC cells. D) The Sankey diagram was utilized to visually depict the trends of data flow between down‐regulated metabolites and various metabolic pathways. E) Metabolomics sequencing on CRC cells before and after ZFPL1 knockdown. F) To assess the activity of ASS1 in CRC cells with ZFPL1 knockdown and overexpression. G) The level of key metabolites in the urea cycle was detected by gas chromatography after ZFPL1 knockdown. In all statistical plots, data are shown as mean ± SEM, Student's t test (Figure 4F,G) were used to determine statistical significance (ns = not significant, **p < 0.01; ***p < 0.001).

Given ASS1's critical role as a rate‐limiting enzyme in arginine biosynthesis and urea cycle regulation,^[^ ^21^ ^]^ we sought to investigate the potential relationship between ZFPL1 and urea cycle modulation in CRC. To address this scientific inquiry, we conducted targeted metabolic profiling following ZFPL1 knockdown in CRC cells. All metabolomic analyses showed that the deletion of ZFPL1 in CRC led to significant changes in the urea cycle metabolites (**Figure** **4**A–E). Detailed metabolic analysis demonstrated that ZFPL1 depletion resulted in substantial alterations in urea cycle metabolites. Specifically, we observed a marked reduction in arginylsuccinic acid, the direct catalytic product of ASS1, along with decreased levels of downstream metabolites including arginine, urea, ornithine, and fumarate. Conversely, upstream metabolites citrulline and aspartate showed significant accumulation (Figure 4G). Notably, these metabolic changes occurred without any detectable alteration in ASS1 enzymatic activity (Figure 4F), suggesting that ZFPL1 can regulate urea cycle function through mechanisms independent of ASS1 activity modulation. Collectively, the above findings suggest that ZFPL1 promotes CRC progression through ASS1‐mediated regulation of the urea cycle.

### 2.4 ZFPL1 Binds to ASS1 and Shields K57 from TRIM33‐Mediated Ubiquitination

**Figure 5:**
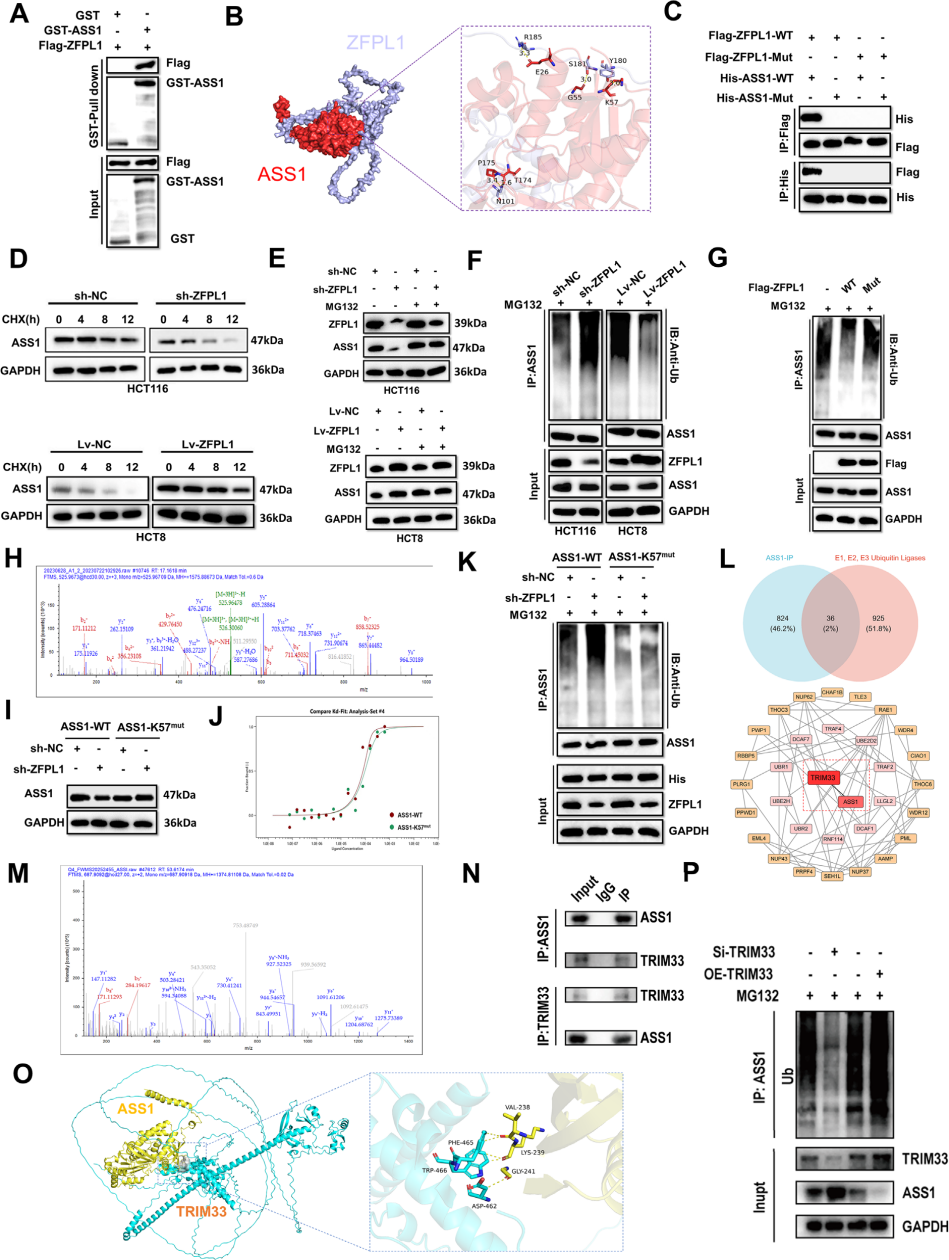
ZFPL1 Binds to ASS1 K57 from TRIM33‐Mediated Ubiquitination. A) GST‐pulldown assays were conducted to illustrate the interaction between ZFPL1 and ASS1 in HEK293T cells transfected with GST, His‐ tagged ASS1, and Flag – tagged ZFPL1. *n* = 3 independent biological replicates. B) Docking was executed using artificial intelligence (AI) technology, uncovering the interaction sites between ZFPL1 and ASS1. C) Co‐IP assay was performed to show the association between ZFPL1‐WT or ZFPL1‐Mut and ASS1‐WT or ASS1‐Mut in HEK293T cells transfected with His‐tagged ASS1‐WT or ASS1‐Mut and Flag‐tagged ZFPL1‐WT or ZFPL1‐Mut. *n* = 3 independent biological replicates. D) CRC cells from various groups were treated with CHX. Subsequently, the cells were collected at specific time points (0, 4, 8, 12 h) for the subsequent western blotting procedure. GAPDH is used for loading control. *n* = 3 independent biological replicates. E) MG132 was used to treat CRC cells from both knocking down and overexpression groups, followed by quantitative analysis using Western blot. GAPDH is used for loading control. *n* = 3 independent biological replicates. F) Following treatment with MG132, immunoprecipitation and western blot analysis were employed to measure the ubiquitination levels of ASS1 in CRC cells where ASS1 was knocked down or overexpressed. GAPDH was utilized as the loading control. *n* = 3 independent biological replicates. G) Co‐IP assays were performed to show the level of ubiquitination of ASS1 in HEK293T cells transfected with Flag‐tagged ZFPL1‐WT or ZFPL1‐Mut. *n* = 3 independent biological replicates. H) Mass spectrometry analysis was employed to identify the specific site of ASS1 ubiquitination. I) Western blot analysis were carried out to determine the level of ASS1 in CRC cells with ZFPL1 knockdown that were transfected with ASS1‐WT or ASS1‐K57^mut^. GAPDH is used for loading control. *n* = 3 independent biological replicates. J) The MST assay was conducted to evaluate the interaction between ASS1‐WT or ASS1‐K57^mut^ and Ub.*n* = 3 independent biological replicates. K) Immunoprecipitation and western blot analysis measured the ubiquitination levels of ASS1 in ZFPL1 knocking down CRC cells transfected with His‐tagged ASS1‐WT or ASS1‐K57^mut^. GAPDH is used for loading control. *n* = 3 independent biological replicates. L) The results of ASS1 IP/MS analysis and the intersection analysis of the E1, E2, and E3 ubiquitin ligase families identified 36 potential ubiquitin ligases that may interact with ASS1. We used STRING to predict the relationships between ASS1 and these 36 proteins online, and identified TRIM33 as the ubiquitin ligase that most significantly affects the ubiquitination level of ASS1. M) The peptide binding to TRIM33 was identified by mass spectrometry of the ASS1 binding protein. N) Co‐IP was used to validate the interaction between ASS1 and TRIM33. *n* = 3 independent biological replicates. O) Docking was executed using artificial intelligence (AI) technology, uncovering the interaction sites between TRIM33 and ASS1. P) Following treatment with MG132, immunoprecipitation and western blot analysis were employed to measure the ubiquitination levels of ASS1 in CRC cells where TRIM33 was knocked down or overexpressed. GAPDH is used for loading control. *n* = 3 independent biological replicates.

To systematically investigate the interaction mechanism between ZFPL1 and ASS1, we adopted a multi‐step experimental approach. Initially, the GST pull‐down assay in HEK293T cells was employed to confirm the direct physical interaction between these two proteins (**Figure** **5**A). Subsequently, we implemented computational structural analysis using artificial intelligence (AI)‐based molecular docking technology, which revealed potential interaction interfaces between ZFPL1 and ASS1 proteins. Detailed analysis of the docking results demonstrated that four specific residues of ZFPL1 (N101, Y180, S181, and R185) form hydrogen bonds with five corresponding residues of ASS1 (T174, P175, K57, G55, and E26). The hydrogen bond distances were measured to be 3.3, 1.6, 3.0, 3.0, and 3.3 Å, respectively (Figure 5B). To validate the computational predictions, we performed site‐directed mutagenesis on both the predicted binding regions of ZFPL1 (ZFPL1‐Mut) and the corresponding interaction domains of ASS1 (ASS1‐Mut). We demonstrated that the physical interaction between ZFPL1 and ASS1 was completely abolished when either protein contained mutations in their respective binding regions, suggesting that these specific sites are essential for maintaining the protein‐protein interaction (Figure 5C). The observed positive correlation between ZFPL1 and ASS1 protein expression levels, independent of ASS1 mRNA regulation, suggests a post‐transcriptional regulatory mechanism underlying their interaction.

Ubiquitination, as one of the crucial regulatory mechanisms of post‐translational modification (PTM), plays a pivotal role in maintaining cellular protein homeostasis.^[^ ^22^ ^]^ Previous studies have established that ubiquitination‐mediated degradation of ASS1 is closely associated with CRC progression.^[^ ^23^ ^]^ Based on existing research evidence, we hypothesize that ZFPL1 may maintain the stability of ASS1 protein through regulating the ubiquitin‐proteasome pathway. To validate this hypothesis, we performed a series of systematic experiments. First, CRC cells with ZFPL1 knockdown or overexpression were treated with cycloheximide (CHX) to inhibit protein synthesis, with or without the proteasome inhibitor MG132. The results showed that ZFPL1 knockdown significantly reduced the half‐life of ASS1 protein compared to control cells, while ZFPL1 overexpression exhibited the opposite effect (Figure 5D). Notably, MG132 treatment effectively reversed ASS1 degradation, suggesting ZFPL1's potential role in inhibiting ubiquitin‐mediated proteasomal degradation of ASS1 (Figure 5E). Subsequent Co‐IP experiments revealed an inverse correlation between ZFPL1 expression levels and ASS1 ubiquitination (Figure 5F). Furthermore, site‐directed mutagenesis of the predicted binding domain on ZFPL1 resulted in a significant increase in ASS1 ubiquitination levels compared to the wild‐type control, indicating that the protein‐stabilizing effect of ZFPL1 on ASS1 is dependent on their specific molecular interaction (Figure 5G).

To identify specific ubiquitination sites on ASS1, we employed liquid chromatography‐tandem mass spectrometry (LC‐MS/MS) analysis of ASS1 protein samples from HCT116 cells under three conditions: ZFPL1 depletion, His‐tagged ASS1 expression, and HA‐tagged ubiquitin (HA‐Ub) expression. Through this comprehensive analysis, lysine 57 (K57) was identified as the exclusive ubiquitination site on ASS1 (Figure 5H). To validate the specific ubiquitination site on ASS1, we first constructed an ASS1‐K57 site mutant (ASS1‐K57^mut^) to investigate the effect of ZFPL1 knockdown on ASS1 protein levels. Comparative analysis between ZFPL1‐knockdown CRC cells and control groups revealed that the ASS1‐K57^mut^ mutant exhibited significantly enhanced protein stability and was no longer affected by ZFPL1 knockdown (Figure 5I). Further investigation demonstrated that mutation at the K57 site did not alter the ubiquitination‐mediated degradation of ASS1 but significantly reduced ZFPL1‐ASS1 interaction strength, as confirmed by microscale thermophoresis (MST) technology (Figure 5J,K). To identify the specific E3 ubiquitin ligase mediating ASS1 ubiquitination and degradation, we intersected LC‐MS/MS‐identified ASS1‐binding proteins with the E1/E2/E3 ubiquitin enzyme families, yielding 36 candidate ubiquitin ligases (Figure 5L). Subsequent STRING database analysis of these candidates revealed TRIM33 as the top‐scoring interactor with ASS1 (Figure 5L), suggesting its highest association probability. Functional validation confirmed TRIM33's role: mass spectrometric analysis of ASS1‐bound proteins detected TRIM33 peptides (Figure 5M), Co‐IP demonstrated their physical interaction in CRC cells (Figure 5N) and the binding energy −2.4 kcal mol^−1^ was calculated by molecular docking of TRIM33 and ASS1 based on AlphaFold3 software (Figure 5O). Crucially, TRIM33 knockdown reduced ASS1 ubiquitination while increasing its expression, whereas TRIM33 overexpression produced the opposite effect (Figure 5P). These findings collectively demonstrate that ZFPL1 inhibits ubiquitin‐proteasome system (UPS)‐mediated degradation of ASS1 through direct protein‐protein interaction, with K57 serving as a critical site mediating ZFPL1‐dependent regulation of ASS1.

### 2.5 ZFPL1 Deficiency Depletes M2 Macrophages and Promotes M1 Polarization in the TME of CRC

**Figure 6:**
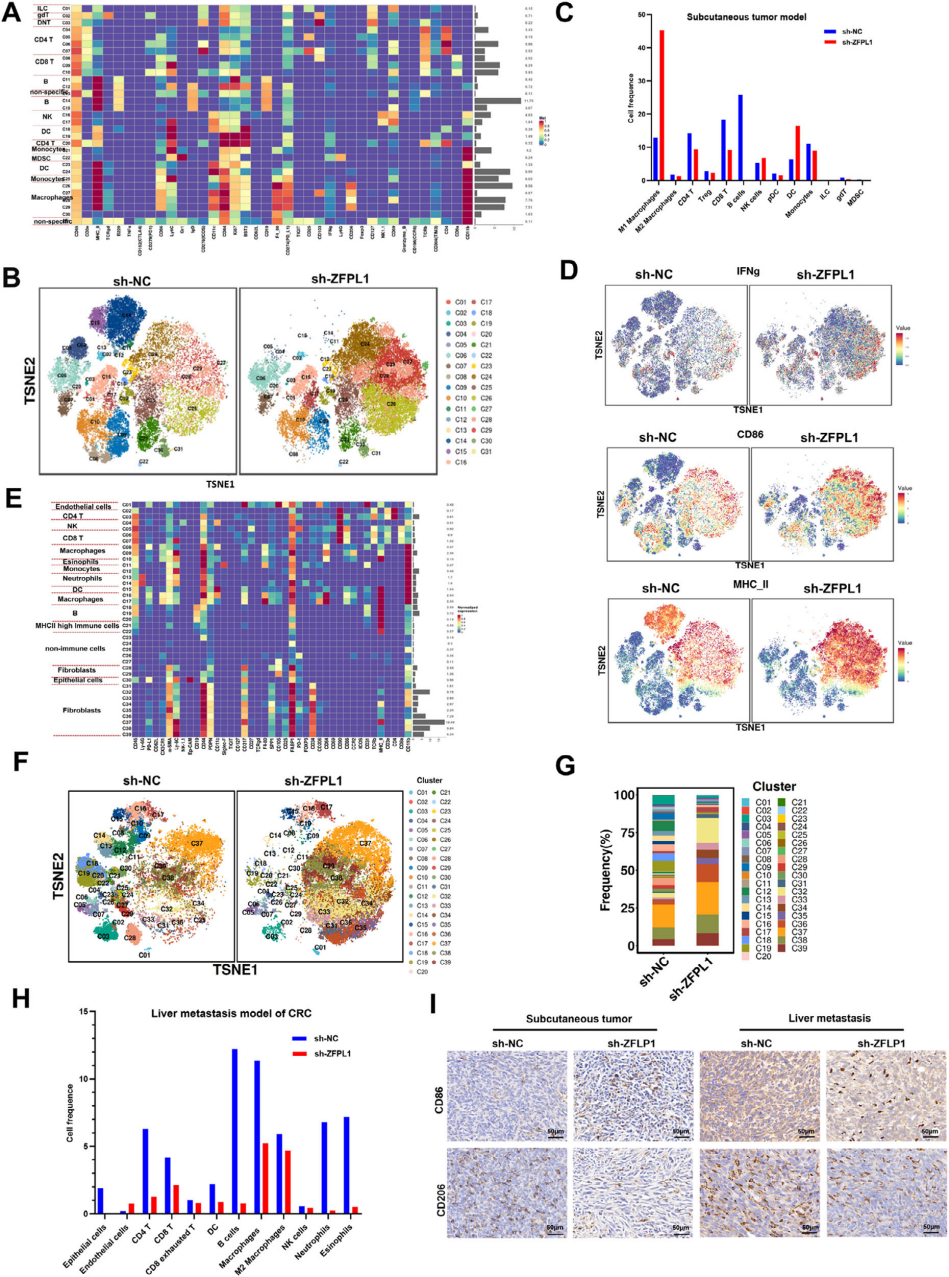
ZFPL1 Deficiency Depletes M2 Macrophages and Promotes M1 Polarization in the TME of CRC. A) Mass cytometry was employed to analyze subcutaneous tumor samples from mice injected with sh‐NC and sh‐ZFPL1. CD45^+^ immune cells that were intact and viable were separated from the selected tissue cells. Aggregation of CD45^+^ immune cells and subgroup annotation were observed in all samples. B,C) The distribution of each cell cluster was presented through a TSNE scatter plot, and the proportion of each cell cluster underwent statistical analysis. D) Specific markers of cell types including CD86, MHC‐II, and IFNg in ZFPL1‐knockdown tumors. E) Mass cytometry was employed to analyze liver metastatic tumor from mice injected with sh‐NC and sh‐ZFPL1. CD45^+^ immune cells that were intact and viable were separated from the selected tissue cells. Aggregation of CD45^+^ immune cells and subgroup annotation were observed in all samples. F,G) The distribution of each cell cluster in liver metastatic tumor was presented through a TSNE scatter plot, and the proportion of each cell cluster underwent statistical analysis. H) In the mass cytometry, the proportions of different cell subgroups in the sh‐NC group and the sh‐ZFPL1 group. I) The expression of CD86 and CD206 in subcutaneous and liver metastatic tumor was detected by IHC (Scale bar, 50 µm).

The aforementioned research has primarily centered on elucidating the impact of ZFPL1 on CRC progression and its underlying molecular mechanisms. However, considering the pivotal role of TME in cancer development and therapeutic response, there remains a critical knowledge gap regarding ZFPL1's immunomodulatory functions within the tumor microenvironment landscape. This gap is particularly significant given the emerging evidence suggesting that tumor‐intrinsic factors can substantially influence immune cell composition and function within the TME, thereby affecting immunotherapy outcomes. To systematically investigate the immunomodulatory effects of ZFPL1 deficiency, we employed a multifaceted experimental approach. Initially, we established a ZFPL1‐knockdown model using shRNA in the murine CRC cell line MC38, followed by comprehensive immune profiling of subcutaneous tumor samples through mass cytometry. Our analytical strategy focused on CD45^+^ immune cells, ensuring the selection of single, viable, and structurally intact cells for subsequent characterization. This rigorous approach yielded the identification of 31 distinct immune cell clusters, each meticulously annotated based on specific marker expression profiles (**Figure** **6**A). Comparative analysis revealed profound alterations in immune cell composition following ZFPL1 knockdown. Specifically, we observed significant decreases in the relative abundance of immunosuppressive cell populations, including M2 macrophages, regulatory T cells (Tregs), and myeloid‐derived suppressor cells (MDSCs) (Figure 6B,C). Notably, the expression of immune molecules, including CD86, MHC‐II, and IFNg, was markedly upregulated in the ZFPL1‐deficient tumors (Figure 6D). Furthermore, mass cytometry analysis of CRC liver metastases identified 39 distinct cell clusters. Results revealed a significant decrease in M2 macrophages within metastatic lesions (Figure 6E–H). Given the consistent observation of macrophage alterations in both subcutaneous tumors and metastatic sites, we performed immunohistochemical staining of subcutaneous tumors and liver metastases. This analysis confirmed reduced M2 macrophage infiltration accompanied by increased M1 macrophage presence in ZFPL1 decreased group (Figure 6I). Beyond its direct tumor‐promoting effects, ZFPL1 deficiency was found to reshape the TME by significantly reducing immunosuppressive cell populations‐M2 macrophages while promoting pro‐inflammatory M1 macrophage polarization, as demonstrated through systematic immune profiling of both subcutaneous and metastatic CRC models.

### 2.6 Sal B Disrupts ZFPL1‐ASS1 Binding at K57 to Sensitize CRC to Immunotherapy

**Figure 7:**
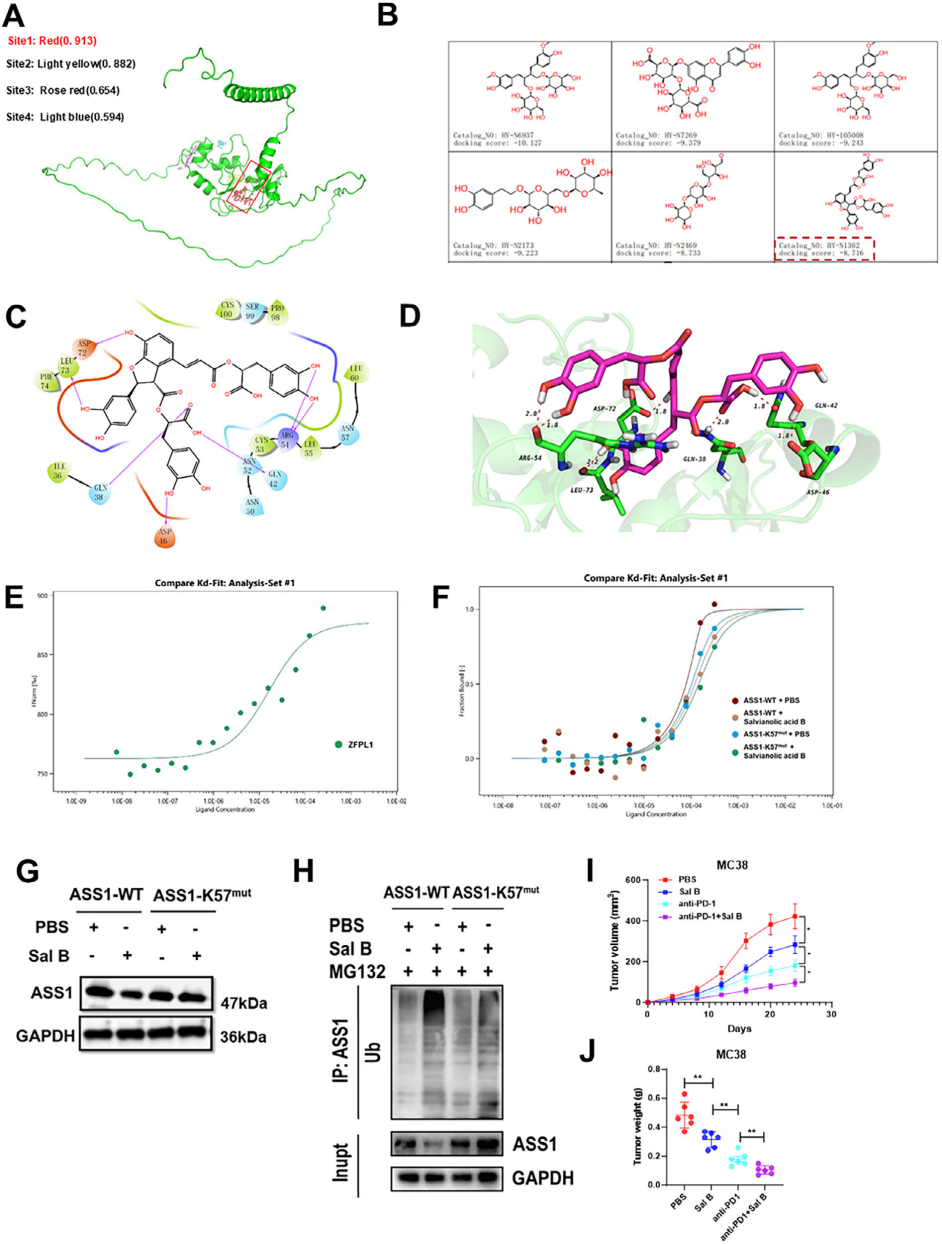
Sal B Disrupts ZFPL1‐ASS1 Binding at K57 to Sensitize CRC to Immunotherapy. A) Diagram illustrating the virtual screening process. B) Chemical structure of Salvianolic acid B. C)The 3D structure of ZFPL1 was utilized to forecast and assess its active site. Virtual screening was centered on Site1, which had the highest – scoring active site, with an emphasis on the key amino acids ILE9, VAL37, and LEU89. D) The binding patterns of Salvianolic acid B with Human ZFPL1 were presented in both 2D and 3D formats. Each hydroxyl group of Salvianolic acid B established three hydrogen bonds with the Human ZFPL1 protein. E) The MST assay was conducted to evaluate the interaction between ZFPL1 and Salvianolic acid B. The determined dissociation constant (Kd) from the fitted binding curve is 8.516 µm. F) The introduction of Sal B significantly decreased the binding rate between ZFPL1 and ASS1, with the ASS1 K57‐mutant group exhibiting a notably lower binding rate compared to the wild‐type group. G) Western blot analysis was conducted to detect the core kinase of ASS1 or ASS1‐K57^mut^ in CRC cells treated with either DMSO or 100 µm Salvianolic acid B. GAPDH is used for loading control. *n* = 3 independent biological replicates. H) Co‐IP detected the ubiquitination level of ASS1 after treatment with DMSO or 100 µm salcinolic acid B in CRC cells transfected with ASS1‐WT or ASS1‐K57^mut^. *n* = 3 independent biological replicates. I) Growth curves of xenograft tumor volumes. *n* = 6 mice per group. J) Graphs of xenograft tumor weights. *n* = 6 mice per group. In all statistical plots, data are shown as mean ± SEM, one‐way ANOVA (Figure 7I) and Student's t test (Figure 7J) were used to determine statistical significance (*p < 0.05; **p < 0.01).

Although functional studies of ZFPL1 have accumulated in recent years, development of small‐molecule therapeutics targeting this protein remains largely unexplored, representing a critical translational gap. To bridge this gap, we performed structural analysis of Human ZFPL1 (ID: AF‐O95159‐F1) using Schrödinger Maestro 11.4. Through 3D modeling and scoring analysis, Site1 was identified as the dominant active site based on superior scoring (**Figure** **7**A). This guided our focus on Human ZFPL1's extracellular Site1 for drug discovery. After docking MCE Library compounds against Human ZFPL1, six candidates were prioritized: (R,R)‐Secoisolariciresinol diglucoside, Luteolin 7‐diglucuronide, Secoisolariciresinol diglucoside, Forsythoside E, Xylotriose, and Salvianolic acid B, following evaluation of docking scores, lipophilicity, and aqueous solubility. Molecular docking assessment revealed Sal B as the strongest ZFPL1 binder (Figure 7B), prompting its selection for further investigation.

Sal B, a naturally occurring polyphenolic compound derived from Salvia species, is renowned for its broad spectrum of biological activities, including anti‐inflammatory, antioxidant, and anti‐fibrotic properties.^[^ ^24^ ^]^ In animal studies, Sal B has been discovered to impede the growth and proliferation of diverse cancer cells, including gastric cancer cells.^[^ ^25^ ^]^ Additionally, it can induce apoptosis and autophagy in cancer cells, suppress their migration and invasion, and thereby inhibit tumor growth and proliferation.^[^ ^24^, ^26^ ^]^ Structural analysis revealed that Sal B interacts with Human ZFPL1 through specific molecular interactions. The 2D and 3D interaction profiles demonstrated that two hydroxyl groups of Sal B form three hydrogen bonds with key residues of Human ZFPL1 (Figure 7C,D). Specifically, these hydroxyl groups interact with THR5 and THR26 residues of the Å chain at distances of 2.8 and 1.7 Å, respectively. Additionally, the carboxyl group of Sal B acts as a hydrogen bond acceptor, forming a bond with the GLN7 residue of the F chain at a distance of 2.2 Å. These interactions suggest a stable and specific binding mechanism between Sal B and ZFPL1. To experimentally validate this binding, we utilized MST technology to measure the dissociation constant (Kd) for the Sal B‐ZFPL1 interaction. The results demonstrated a strong binding affinity, with a Kd value of 8.516 µm (Figure 7E). Furthermore, we observed that the addition of Sal B significantly reduced the binding rate between ZFPL1 and ASS1, with the ASS1‐K57^mut^ group showing a notably lower binding rate compared to the wild‐type group (Figure 7F). These findings suggest that Sal B may exert its effects by disrupting the ZFPL1‐ASS1 interaction at the K57 site. Subsequent western blot analysis corroborated these results, showing a reduction in wild‐type ASS1 protein levels upon Sal B treatment, while the levels of the K57 mutant remained unchanged (Figure 7G). Co‐IP analysis showed that Sal B treatment with enhanced ubiquitination but decreased expression of the wild‐type ASS1 protein (Figure 7H). This further supports the hypothesis that Sal B inhibits the binding of ZFPL1 to ASS1 at the K57 site. To evaluate the therapeutic potential of Sal B in vivo, we conducted experiments in C57BL/6 mice injected with MC38 cells. On day 7, mice were treated with Sal B and anti‐PD‐1, and the results revealed a significant reduction in tumor volume and weight in the Sal B+anti‐PD‐1 group compared to the anti‐PD‐1 group (Figure 7I,J). These findings collectively indicate that Sal B enhances the sensitivity of anti‐PD‐1 immunotherapy by suppressing ZFPL1 activity. This not only validates its potential as a promoter of cancer immunotherapy but also highlights its translational potential in CRC treatment. Future investigations should focus on elucidating the precise molecular mechanisms of Sal B's action and exploring its synergistic potential with other immunotherapeutic modalities.

## 3 Discussion

ZFPL1, a gene located on chromosome 11q13, encodes a 310‐amino acid protein with significant biological implications.^[^ ^27^ ^]^ Previous studies have established that ZFPL1 functions as a conserved integral membrane protein, demonstrating crucial interactions with the cis‐Golgi matrix protein GM130. These interactions are essential for maintaining the structural and functional integrity of the cis‐Golgi apparatus.^[^ ^28^ ^]^ Emerging evidence has highlighted the oncogenic potential of ZFPL1 in various malignancies. Research conducted by Mei‐Juan Cai et al. revealed that ZFPL1 knockdown in endometrial cancer cells resulted in inhibited cell proliferation and downregulation of p‐Akt308 and p‐Akt473 expressions, while simultaneously upregulating PTEN protein levels. Conversely, overexpression of both ZFPL1 and its transmembrane domain‐deficient variant (ZFPL1ΔTMD) promoted cellular proliferation and activated p‐Akt308 and p‐Akt473 expressions.^[^ ^29^ ^]^ Despite these advancements, the precise role of ZFPL1 in CRC pathogenesis remains poorly understood. Our study, employing scRNA‐seq analysis, revealed significant enrichment of ZFPL1 in malignant CRC cells. Through comprehensive in vitro and in vivo experiments, we have demonstrated that ZFPL1 plays a pivotal role in enhancing CRC proliferation, invasion, and migration. Particularly noteworthy is our discovery of ZFPL1's critical involvement in CRC liver metastasis. Our findings indicate that ZFPL1 is not only enriched at metastatic sites but also that its inhibition significantly attenuates CRC liver metastasis in vivo. These findings substantially expand our understanding of ZFPL1's role and molecular mechanisms in tumor biology, particularly in the context of CRC liver metastases.

Cancer cells undergo metabolic reprogramming to efficiently utilize nitrogen and carbon sources for the synthesis of macromolecules essential for tumor proliferation and growth.^[^ ^30^, ^31^ ^]^ The urea cycle, a critical biological pathway responsible for converting nitrogenous metabolites into urea, plays a pivotal role in nitrogen waste disposal. Dysregulation of the urea cycle, often caused by specific alterations in urea cycle enzymes, is a well‐documented metabolic disorder in cancer.^[^ ^32^ ^]^ For instance, the tumor suppressor protein p53 inhibits tumor growth by suppressing urea production and ammonia clearance. Loss of p53 function leads to increased urea production, which promotes pyrimidine synthesis and, consequently, cancer progression.^[^ ^33^ ^]^ Furthermore, impaired urea cycle activity not only contributes to carcinogenesis but also plays a crucial role in preventing ASS1 ubiquitination and degradation, thereby enhancing urea cycle activity and accelerating CRC progression. Our study has elucidated the intricate interplay between ZFPL1 and the urea cycle in CRC, highlighting its significance in tumor metabolism.^[^ ^34^ ^]^ Ubiquitination, a key post‐translational modification, regulates protein stability and function. Dysregulation of ubiquitination is frequently observed in various cancers, underscoring its critical role in tumor progression.^[^ ^35^ ^]^ Previous studies have demonstrated that ASS1 is overexpressed in CRC and functions as an oncogene, promoting cancer cell growth and metastasis. However, the precise molecular mechanisms underlying ASS1 ubiquitination remain poorly understood. Although some studies suggest that LOC113230 can inhibit arginine synthesis by enhancing ASS1 ubiquitination and degradation, the specific ubiquitination sites on ASS1 have not been identified.^[^ ^36^ ^]^ In this study, we demonstrated that ZFPL1 competitively binds to ASS1, thereby inhibiting its ubiquitination and subsequent degradation. Utilizing mass spectrometry sequencing, we identified that ZFPL1 binding to ASS1 prevents ubiquitination at the K57 site. Furthermore, through Co‐IP and mass spectrometry analyses, we found that the E3 ubiquitin ligase TRIM33 specifically interacts with and mediates the ubiquitination of ASS1. These findings provide important insights into the regulatory mechanisms of ASS1 and open new avenues for investigating ASS1‐related pathways.

The aforementioned research focused on the proliferation and metastasis mechanisms of CRC. However, beyond the cancer cells themselves, the TME of liver metastases exhibits a highly immunosuppressive phenotype that promotes highly dynamic dissemination.^[^ ^37^ ^]^ To explore how cancer cells colonize the liver and form metastatic niches, we employed mass cytometry to compare subcutaneous tumors and metastatic lesions, investigating the influence of ZFPL1 on the composition and function of immune cells within the TME of liver metastases. Our results revealed that ZFPL1 deficiency significantly reduces immunosuppressive cell populations—particularly M2 macrophages—while promoting polarization toward proinflammatory M1 macrophages, thereby remodeling the TME. Consistent with previous studies, we found that M2 macrophages enhance cancer cell invasiveness; for instance, they activate the CD36‐dependent epithelial–mesenchymal transition (EMT) pathway in CRC, thereby promoting tumor aggression.^[^ ^38^ ^]^ Additionally, M2 macrophages secrete anti‐inflammatory cytokines such as IL‐10 and TGF‐β, which suppress T cell‐mediated antitumor immunity and disrupt immune cell crosstalk, fostering an immunosuppressive microenvironment.^[^ ^39^ ^]^ Therefore, M2‐polarized TAMs create a TME that supports tumor growth and amplifies metastasis. Based on the above results, our study provides further evidence supporting this concept. However, further research is needed to determine which cytokines are recruited by ZFPL1 to attract M2 macrophages.

Another significant contribution of this study is the identification of small molecule inhibitors targeting ZFPL1. Through structural virtual screening, we identified Sal B as a potent ZFPL1 inhibitor capable of attenuating CRC progression. Sal B, a bioactive compound derived from traditional herbal medicine, exhibits a wide range of pharmacological properties and has been used in the treatment of various diseases, including liver, kidney, brain, heart, and vascular disorders, as well as cancer and diabetes.^[^ ^40^, ^41^ ^]^ In HCT116 colon cancer cells, Sal B suppresses the Akt/mTOR signaling pathway, leading to the upregulation of autophagy‐related proteins LC3‐II and Atg5, thereby inducing autophagy and inhibiting cancer cell proliferation.^[^ ^42^ ^]^ In our study, we not only confirmed the binding of Sal B to the active site of ZFPL1 but also demonstrated through MST experiments that Sal B significantly reduces the binding affinity between ZFPL1 and ASS1. In vivo experiments further validated that the combination of Sal B with a PD‐1 monoclonal antibody synergistically enhances the anti‐CRC effect, providing a promising therapeutic strategy for CRC treatment.

While this study establishes a pivotal role for ZFPL1 in promoting colorectal cancer progression and metastasis through ASS1 stabilization, urea cycle reprogramming, and immunomodulation, several limitations remain to be addressed. Our clinical findings, derived primarily from a single institutional cohort of 80 patients, require validation in larger multicenter populations to ensure broader applicability. Mechanistically, although we demonstrate that ZFPL1 enhances arginine biosynthesis through mass action via ASS1 protein accumulation, further detailed investigations—such as metabolic flux analysis and in vivo validation of ASS1 degradation in ZFPL1‐overexpressing models—are needed to fully elucidate the mechanistic underpinnings. Beyond the urea cycle, it remains plausible that ZFPL1 may influence other metabolic pathways such as the TCA cycle or nucleotide synthesis, warranting future exploration. Additionally, while ZFPL1 loss shifted macrophage polarization from M2 to M1, the specific cytokines and signals mediating this effect remain uncharacterized. Although we propose a potential link between ZFPL1 and immunotherapy sensitivity, direct clinical correlations with chemotherapy or immune checkpoint inhibitor resistance await further investigation. The therapeutic potential of salvianolic acid B also necessitates additional pharmacokinetic and safety profiling to evaluate its bioavailability and off‐target effects. Finally, this work focuses on local tumor microenvironment mechanisms, leaving open questions regarding systemic or epigenetic effects of the ZFPL1‐ASS1 axis. Addressing these limitations in future studies will be essential to advancing ZFPL1‐targeted therapeutic strategies and enhancing their translational relevance.

## 4 Conclusion

**Figure 8:**
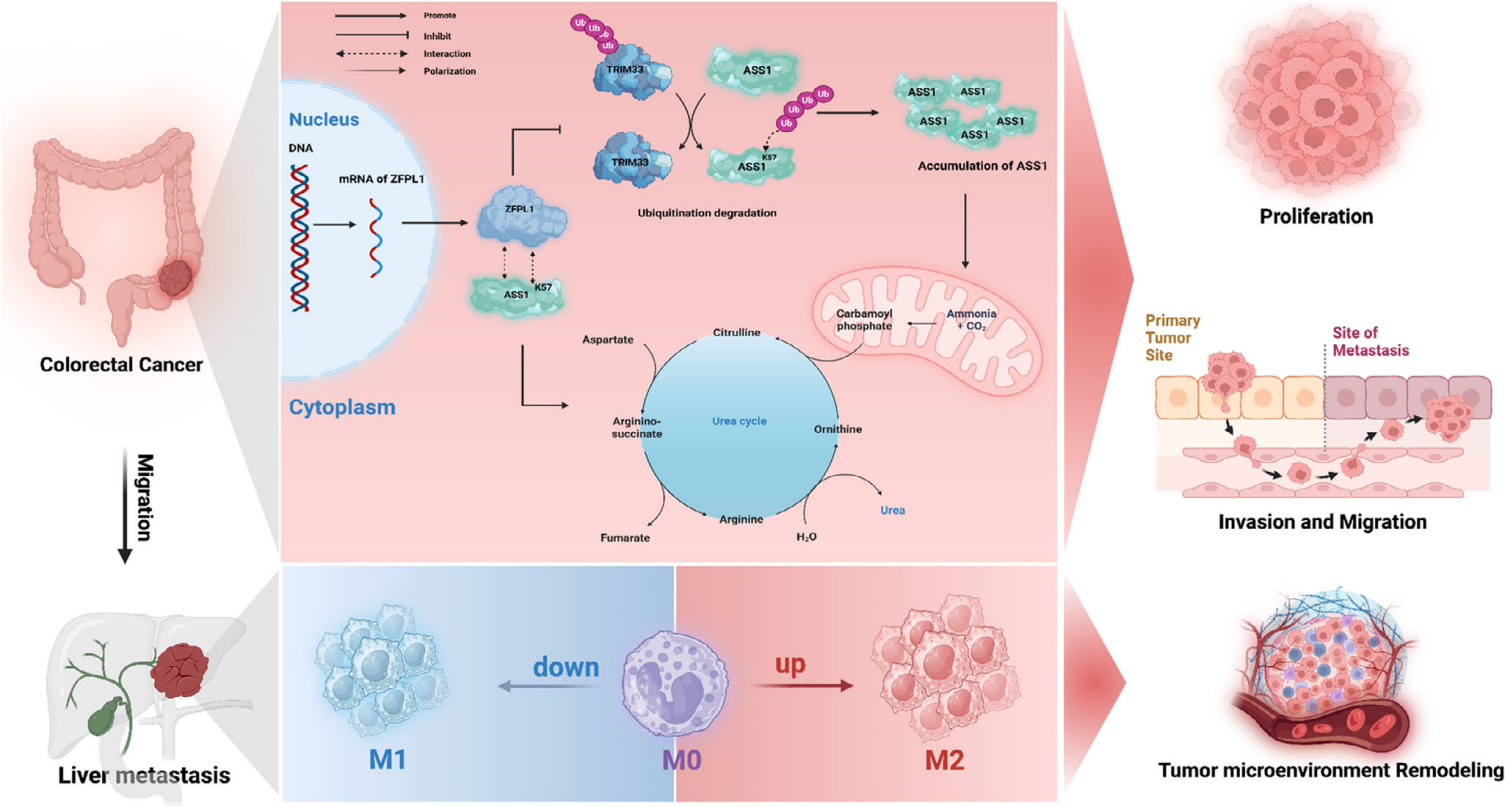
Pattern diagram. ZFPL1 Orchestrates CRC Progression through ASS1 Stabilization‐driven Urea Cycle Activation and M2 Macrophage‐mediated Metastatic Colonization (Created with BioRender.com).

ScRNA‐seq reveals ZFPL1‐specific enrichment in malignant CRC cells. Mechanistically, ZFPL1 stabilizes ASS1 by blocking K57 ubiquitination, activating urea cycle metabolism to drive progression. ZFPL1 inhibition reprograms the TME by suppressing M2 macrophages polarization and promotion M1 macrophages, thereby inhibiting CRC liver metastasis. Computational screening identifies Sal B as a potent ZFPL1 inhibitor with efficacy in CRC models. Collectively, ZFPL1 thus emerges as a metabolic‐immune regulator enabling novel therapeutic strategies in CRC (**Figure** **8**).

## 5 Experimental Section

### Single‐Cell RNA Data Analysis

ScRNA‐seq data from the GEO database (accession numbers: EMTAB8107, GSE146771, and GSE164522) were utilized to analyze the differential expression of ZFPL1 mRNA in CRC tissue samples. Cells were selected for further analysis if they met the following criteria: unique molecular identifier (UMI) counts ranging from 3000 to 40 000 and mitochondrial gene content below 10% of the total UMI count. UMI counts were normalized and converted to gene expression levels using the formula LOG2 (UMI + 1).

### Cells and Cell Culture

Mouse CRC cells (MC38) and human CRC cells (HCT116, HCT8, SW480, and Lovo) were obtained from the Type Culture Collection Bank. Cells were cultured in RPMI Medium 1640 or DMEM supplemented with 10% fetal bovine serum (Gibco, USA) and 1% antibiotic mixture (penicillin and streptomycin).

### Collection of Patients and Tissue Specimens

In accordance with the Helsinki Declaration, all patients were provided with detailed information about the study and provided informed consent. Primary CRC tissues, liver metastasis tissues, and adjacent normal tissues were collected from patients undergoing surgical resection or preoperative puncture at the First Affiliated Hospital of Nanjing Medical University or the Affiliated Cancer Hospital of Zhengzhou University & Henan Cancer Hospital. The study was approved by the Ethics Committee of Nanjing Medical University and the Affiliated Cancer Hospital of Zhengzhou University & Henan Cancer Hospital (2023‐SRFA‐248, 2020051307‐022). All tumor specimens were validated and classified by experienced clinicians.

### qRT‐PCR and RNA Extraction

Total RNA was extracted from tissues and cells using the Cell/Tissue Total RNA Isolation Kit (Vazyme, China). RNA was reverse‐transcribed into cDNA using a reverse transcription kit (Vazyme, China). Primer sequences are listed in Table S2 (Supporting Information). GAPDH was used as an internal control to normalize mRNA expression levels.

### Cell Transfection

ZFPL1 expression was inhibited in human and mouse CRC cell lines using shRNA (Genechem, China). Briefly, 1 × 10^5^ cells were seeded per well in a 6‐well plate with 2 mL of culture medium and incubated for 24 h. Subsequently, 40 µL of polybrene (Sigma–Aldrich, USA) and the appropriate amount of virus were added to the medium. After 12–16 h of incubation, cells were cultured in standard medium supplemented with puromycin (Beyotime, China) for selection. Transfection efficiency was evaluated using qRT‐PCR and Western blotting.

### Cell Proliferation Assay

Cells were seeded into 96‐well plates at a density of 1000 cells per well in 100 µL of medium and incorporated 10 µL of CCK‐8 solution (RiboBio, China) was utilized. A microplate reader was used to measure cell absorbance (OD) at 450 nm at 1, 2,3, 4, and 5 days of culture following the guidelines provided by the manufacturer (Synergy, USA). Cell proliferation was also assessed using the EdU DNA Cell Proliferation Kit (RiboBio, China). In a 24‐well plate, each well was populated with 50 000 cells. Post‐regular culture, cells were exposed to 50 mmol L^−1^ EdU solution for a period of 2 h and subsequently fixed with 4% paraformaldehyde. In accordance with the kit's instructions, the cellular strains with Apollo Dye Solution and DAPI, followed by imaging and enumeration using an Olympus FSX100 microscope (Olympus, Japan) were treated. For the colony formation assay, 1000 CRC cells were seeded into each well of a 6‐well plate. After 10 days, cells were fixed with methanol and stained with Giemsa (Beyotime, China). Colonies were imaged and counted.

### Transwell Assay

CRC cells were seeded into the upper chamber of a Transwell apparatus in 200 µL of serum‐free RPMI 1640 medium (Gibco, USA). For invasion assays, the basement membrane was pre‐coated with Matrigel (BD Biosciences, USA). The lower chamber was filled with culture medium containing 10% fetal bovine serum. After incubation, cells in three distinct areas were counted and imaged.

### Wound Healing Assay

CRC cells were seeded uniformly in a 6‐well plate, and a scratch was created in the confluent cell layer using a 20‐µL pipette tip. The controlled elimination process aimed to segregate debris and floating cells at the well's base. Subsequently, a serum‐free medium was introduced, and the plate was subjected to a 37 °C incubation. The width of the scratch was measured using an inverted microscope, and images were captured at 0 and 48 h. This experimental procedure was repeated thrice to assess both the initial wound width and the extent of cell migration.

### Immunohistochemistry and Immunofluorescence

Paraffin‐embedded sections were deparaffinized and rehydrated for immunohistochemical analysis. Peroxidase activity was blocked using 3% hydrogen peroxide (Beyotime, China). Tissue sections were incubated overnight at 4 °C with primary antibodies (CD8, CD86, and CD206), followed by incubation with a biotinylated secondary antibody and streptavidin‐horseradish peroxidase (Beyotime, China). For immunofluorescence, samples were fixed with 4% paraformaldehyde for 20 min, permeabilized with 0.05% Triton X‐100 for 5 min, and blocked with 10% donkey serum in PBS for 1 h. Primary and secondary antibodies were incubated for 1 h each at room temperature. Samples were mounted using fluorescent mounting medium. Time of 24 h in advance, CRC cells are passaged, and the cells were evenly inoculated on sterile cover glasses placed in a six‐well plate. After overnight incubation, the cell confluence was allowed to reach 70%. After washing the cells with PBS, they were fixed with 4% paraformaldehyde for 10 min. After washing the cells with PBS again, a blocking solution (Beyotime, China) was added and incubated for 60 min. The primary antibody was then added after dilution and incubated overnight at 4 °C. After 24 h, the cells were washed three times with TBST, and then fluorescent secondary antibodies (Beyotime, China) were added and incubated for 1 h. Images were captured using a Zeiss confocal microscope (Germany). The antibodies used in this research are specified in Table S3 (Supporting Information).

### Western Blotting

Whole cell proteins were extracted from the cells using RIPA lysis buffer (NCM, China) supplemented with protease and phosphatase inhibitors (Beyotime, China). After protein extraction, the appropriate loading buffer (Beyotime, China) was added, heated in 99 °C water bath for 5 min, and stored at −80 °C. The proteins were separated by 10% SDS‐PAGE (NCM, China), and transferred to PVDF membranes (Millipore, USA) as per the manufacturer's operating manual. After blocking the membranes for 30 min with blocking buffer (NCM, China), the membranes were incubated with primary antibodies for a whole night at 4 °C. The ECL signals were seen using an ECL Kit (NCM, China) following a 1 h incubation period with the corresponding secondary antibodies and three TBST‐buffered saline washings every 10 min. Antibodies used in this study are listed in Table S3 (Supporting Information).

### RNA Sequencing

To investigate the global gene expression changes upon ZFPL1 suppression, sh‐ZFPL1 and control HCT116 cells were lysed using Trizol, and RNA sequencing was performed. Data analysis was conducted by Berry Genomics (Beijing, China).

### Metabolomics Sequencing and Analysis

The metabolite extraction protocol was performed as follows: Cellular residues were subjected to ultrasonic treatment in 1 mL of a pre‐cooled acetonitrile/methanol/water solution. The mixture was maintained in an ice bath for 1 h, followed by incubation at −20 °C for an additional hour to ensure metabolite stabilization. The samples were then centrifuged at 14 000 g for 20 min at 4 °C, and the supernatant was collected for subsequent LC‐MS analysis. Metabolomic profiling was conducted using a UPLC‐ESI‐Q Exactive Plus mass spectrometer (Thermo Scientific). To maintain analytical consistency and accuracy, all organic solvents, including acetonitrile (ACN), were of HPLC grade. Quality control measures included performing blank injections after every six sample analyses throughout the analytical sequence. For data processing, raw mass spectrometry data were analyzed using MS‐DIAL (Mass Spectrometry Data Analysis Tool), which enabled comprehensive data processing including peak alignment, retention time correction, and peak area quantification. Metabolite identification was achieved through comparison of MS/MS spectra against both public databases and an in‐house library of authentic metabolite standards. To elucidate the biological significance of identified differential metabolites, pathway analysis was performed using the KEGG database (Kyoto Encyclopedia of Genes and Genomes; http://www.kegg.jp). Statistical significance of pathway enrichment was determined using Fisher's exact test, with multiple testing correction implemented through the False Discovery Rate (FDR) method. Pathways with corrected p‐values < 0.05 were considered statistically significant.

### Gas Chromatography‐Mass Spectrometry

Analyses were performed using gas chromatograph coupled to a mass spectrometer (GC–MS; Thermo Scientific Polaris Q Trace GC Ultra, Switzerland) using a capillary column RTX‐1701 (30 m × 0.32 mm ID, 0.25 µm, Restek, USA). Xcalibur Software was used to process the data. The injector temperature was set at 250 °C, and 1 µL was injected with a split ratio of 200:1. Ultra‐high‐purity helium (99.9995%) was used as carrier gas at constant flow rate of 1.2 mL min^−1^. The oven temperature program was as follow: 2 min at 150 °C, ramp at 35 °C min^−1^ until 230 °C, ramp at 100 °C min^−1^ to 250 °C, and hold for 2 min. Total elution time was 9 min. The transfer line and ion source temperatures were 300 and 230 °C, respectively. Mass detection was performed in full scan (range *m*/*z* 230–239); non‐, mono‐, and di‐labeled‐ and internal standard‐urea *tert*‐butyldimethylsilane (TBDMS) were identified and quantified at *m*/*z* 231, 232, 233, and 236, respectively. For each matrix, a blank and five different concentrations of standard solutions were used in triplicates.

### Immunoprecipitation and Mass Spectrometry

HEK293T cells were transfected with the specified plasmid and incubated for 24 h. Cells were lysed in IP lysis buffer containing protease and phosphatase inhibitors for 30 min. Lysates were analyzed by western blotting using an anti‐FLAG antibody (Sigma–Aldrich, USA) and visualized using an Odyssey Fc Imaging System (LI‐COR Biosciences, USA). Protein bands were quantified using Image Studio Lite software (LI‐COR Biosciences). Statistical analysis was performed using one‐way ANOVA (p < 0.05). Lysates were also subjected to mass spectrometry analysis using a TripleTOF 5600 system (AB Sciex, USA).

### Co‐Immunoprecipitation (Co‐IP)

Co‐IP assay was performed as we described before.^[^ ^43^ ^]^ Briefly, proteins were immunoprecipitated by using an IP kit (Termo Fisher Scientifc, CA, USA) according to the manufacture's protocols and then assessed by immunoblotting.

### GST Pull‐Down Assay

Purified recombinant ZFPL1 protein (15 µg) was incubated with glutathione agarose gel at 4 °C for 1 h, followed by washing with binding buffer. Purified recombinant ASS1 protein (15 µg) was added and incubated for another hour under the same conditions. The protein complex was washed and eluted using elution buffer. Eluted proteins were analyzed by SDS‐PAGE and Western blotting. GST (glutathione S‐transferase) protein was used as a negative control.

### Microscale Thermophoresis (MST)

The use of MicroScale Thermophoresis (MST) technology to assess the binding affinity between ZFPL1 and ASS1 or Salvianolic acid B involves both conventional capillaries and the Monolith NT.115 produced by NanoTemper Technologies GmbH of Germany. The technical process involves fluorescent labeling of ZFPL1, followed by mixing the labeled ZFPL1 with varying concentrations of ASS1 or Salvianolic acid B in a buffer solution (pH 7.4, containing 1 mm MgCl2, 3 mm KCl, 150 mm NaCl, and 0.05% Tween‐20) to achieve optimal analysis of molecular interactions. The purpose of this step is to quantify the binding affinity between ZFPL1 and ASS1 or Salvianolic acid B. During the experiment, the experimental data were fitted and analyzed by MO Affinity Analysis v2.1.3 software to obtain specific values of the binding constant. This analysis software can process MST experimental data to accurately assess the strength of interaction between proteins and ligands.

### Prediction of Potential Allosteric Sites of ZFPL1 and Virtual Screening

Potential allosteric sites in the ZFPL1 protein structure were predicted using AlphaFold. Virtual screening was performed across two commercial chemical libraries, each containing over 67 000 compounds. The top five compounds with the highest scores from the MCE screening were selected for further experimental evaluation.

### Animal Model

All animal experiments were conducted in accordance with the institutional ethical guidelines and were approved by the Animal Care and Use Committee of Nanjing Medical University (IACUC‐2404094). C57BL/6 mice were maintained under specific pathogen‐free (SPF) conditions throughout the study period. At the experimental endpoint, euthanasia was performed by cervical dislocation following established protocols. For the subcutaneous tumor model, C57BL/6 mice (*n* = 6 per group) received right groin injections of 1 × 10^6^ MC38 cells that had been transfected with ZFPL1 shRNA (sh‐ZFPL1). Following the experimental protocol, mice were sacrificed for subsequent immunohistochemical analysis of liver protein expression. The CRC liver metastasis model was established through splenic transplantation of MC38 cells. Briefly, five‐week‐old male C57BL/6 mice were anesthetized and subjected to aseptic surgical procedures. After making a left flank incision, the spleen was carefully exteriorized and stabilized using sterile gauze. A suspension containing 1 × 10^6^ MC38 cells in 50 µL PBS was injected into the splenic pulp using a 26‐gauge needle. Following cell inoculation, the spleen was repositioned, and the abdominal wall was closed with non‐absorbable sutures. For therapeutic evaluation, tumor‐bearing mice were randomly allocated into four experimental groups (*n* = 6 per group): PBS control, Sal B alone, anti‐PD‐1 alone, and Sal B + anti‐PD‐1 combination. The treatment regimen consisted of intraperitoneal administration of 6.6 mg kg^−1^ anti‐PD‐1 (Bioxcell, USA) for the combination group. Sal B (MCE, USA) was administered at 10 mg kg^−1^, with the initial dose given on day 8 followed by twice‐weekly injections. An alternative dosing schedule involved Sal B administration starting on day 7 and continuing every other day for two weeks.

### Mass Cytometry

The detailed experimental methods are shown in previous article.^[^ ^44^ ^]^

### Statistical Analysis

Most of the analyses in this study were performed by Graphpad Prism 10.0 with p‐value of 0.05 for statistical significance. Data are presented as mean ± SEM. Student's t test was used to analyze the differences between the two sample groups. One‐way ANOVA was used to analyze the differences between three or more sample groups. Detailed statistical analysis is provided in the respective figure legends.

### Ethics Approval and Consent to Participate

This study, which involves human participants, received approval from the Ethics Committee of Nanjing Medical University and the Affiliated Cancer Hospital of Zhengzhou University & Henan Cancer Hospital (2023‐SRFA‐248, 2020051307‐022). All in vivo animal experiments were approved by the Committee on the Ethics of Animal Experiments of Nanjing Medical University (IACUC‐2404094).

## Conflict of Interest

The authors declare no conflict of interest.

## Author Contributions

X.Q., C.X., B.Z., H.C., Z.L., and L.L. contributed equally to this work. This manuscript features six primary authors: Dr. X.J.Q., C.X.X., B.L.Z., H.S.C., Z.Q.L., and L.L. Their responsibilities encompassed study design, executing a portion of the experiments, and drafting the manuscript. Additionally, five corresponding authors, Dr. X.P.H., J.X.Z., W.W.T., H.B.Y., and Y.Y.L., played integral roles in study design, data interpretation, editing, and critically revising the manuscript. Other contributors participated in conducting sections of the experiments and interpreting the data. All authors thoroughly reviewed and endorsed the final manuscript.

## Supporting information



Supporting Information

## Data Availability

The data that support the findings of this study are available from the corresponding author upon reasonable request.
