# Supplementary material for: ZFPL1 Promotes Colorectal Cancer Progression by Stabilizing ASS1 to Drive the Urea Cycle and M2 Macrophage‐Mediated Metastatic Colonization
Source: Adv Sci (Weinh). 2025 Nov 11;12(46):e05291. doi: 10.1002/advs.202505291 (PMC12697865; doi:10.1002/advs.202505291)
Supplement: Supplementary file 1 — Supporting Information [file ADVS-12-e05291-s001.docx]

**Supplementary figures**

**Figure S1:**

(A-B) The heat map shows the specific genes expressed by the cell clusters of figure 1A.

(C-D) According to the definition of classification of specific gene markers using UMAP plot and dot plot, GSE146771 results indicated that a total of 13 cell clusters in CRC.

(E) The dot plot shows the specific genes that define different cell clusters.


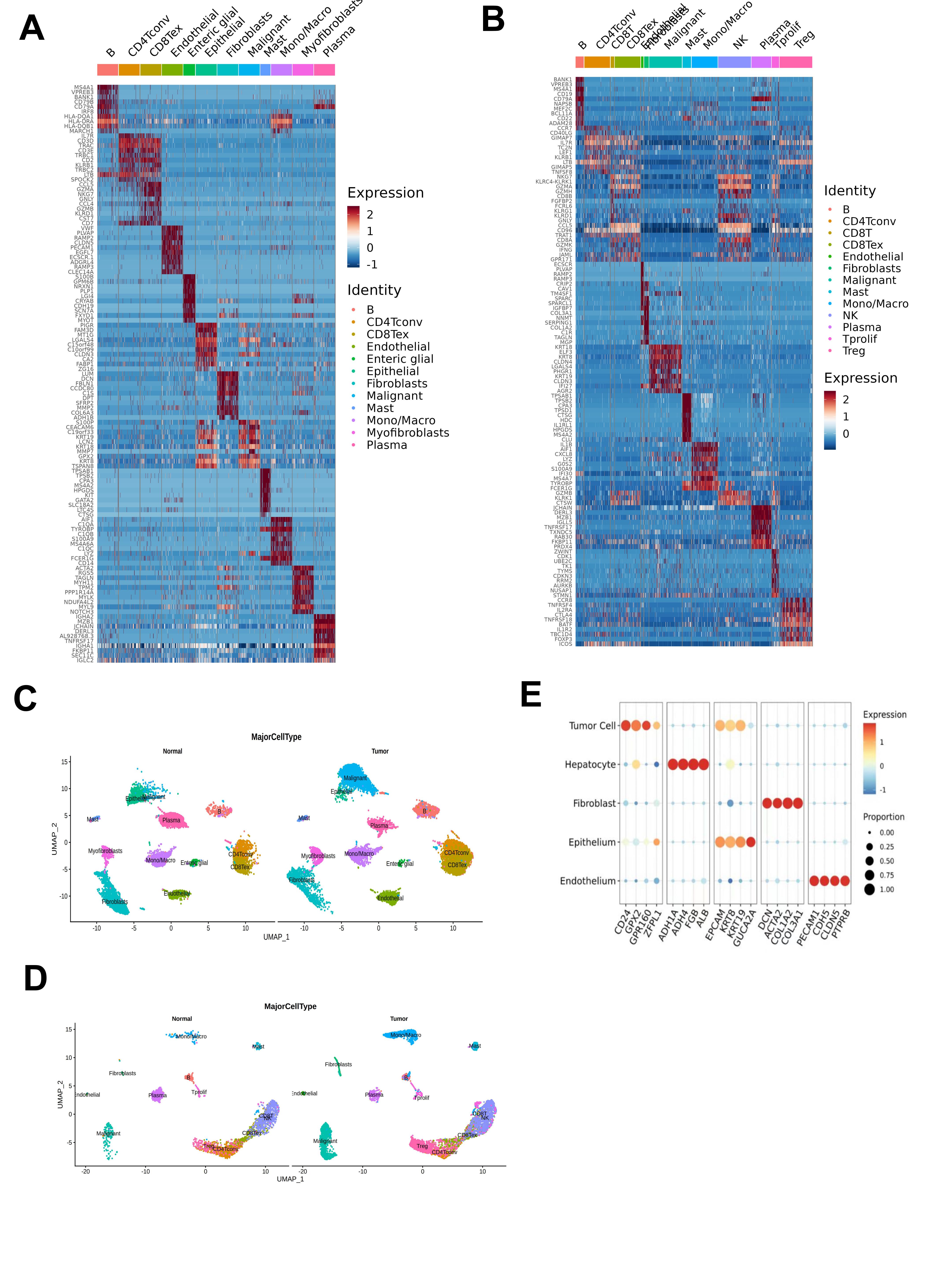


**Figure S2:**

(A-B) The single-cell RNA sequencing results of 5 samples of one CRC liver metastasis patient samples revealed the existence of 5 major cell clusters within CRC, as defined by the UMAP plot and dot plot for the classification of specific genetic markers.

(C-E) According to the classification of specific genetic markers defined by UMAP and dot plots, the sequencing results revealed 13 minor cell clusters within the myeloid cells, which were distributed across primary tumors, adjacent normal tissues of primary tumors, liver metastases, adjacent normal tissues of metastases, and blood.

(F-G) According to the classification of specific genetic markers defined by UMAP and dot plots, the sequencing results revealed 8 minor cell clusters within the stromal cells.

(H) The violin diagram measures the EMT. Score of specific genes in the EMT pathway.

(I) The dot plot illustrates the expression of ZFPL1 in tissues.


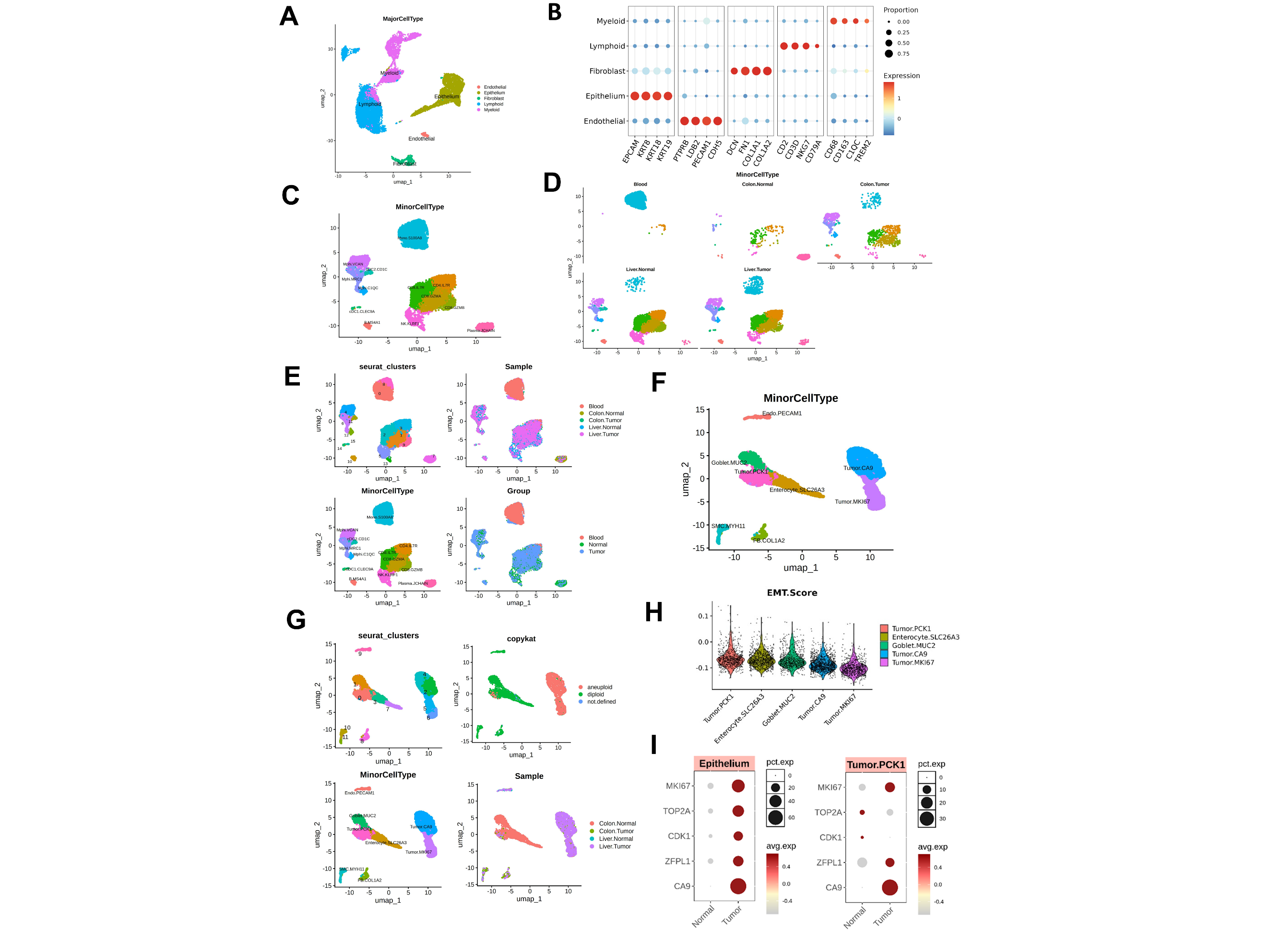


**Figure S3:**

(A) Time-dependent ROC curve analysis of H-score of ZFPL1 in CRC patients (n=80).

(B) Kaplan-Meier analysis of the correlation between ZFPL1 expression and disease-free survival (DFS) in patients (H-score cutoff value =126.3, n=80).

(C) Kaplan-Meier analysis of the association between ZFPL1 expression and overall survival (OS) in patients (H-score cutoff value =126.3, n=80).

(D) Kaplan-Meier analysis of the correlation between ZFPL1 expression and overall survival of colon cancer in Kaplan-Meier Plotter (n=1061).

(E) Kaplan-Meier analysis of the correlation between ZFPL1 expression and recurrence free survival (RFS) of colon cancer in Kaplan-Meier Plotter (n=1336).


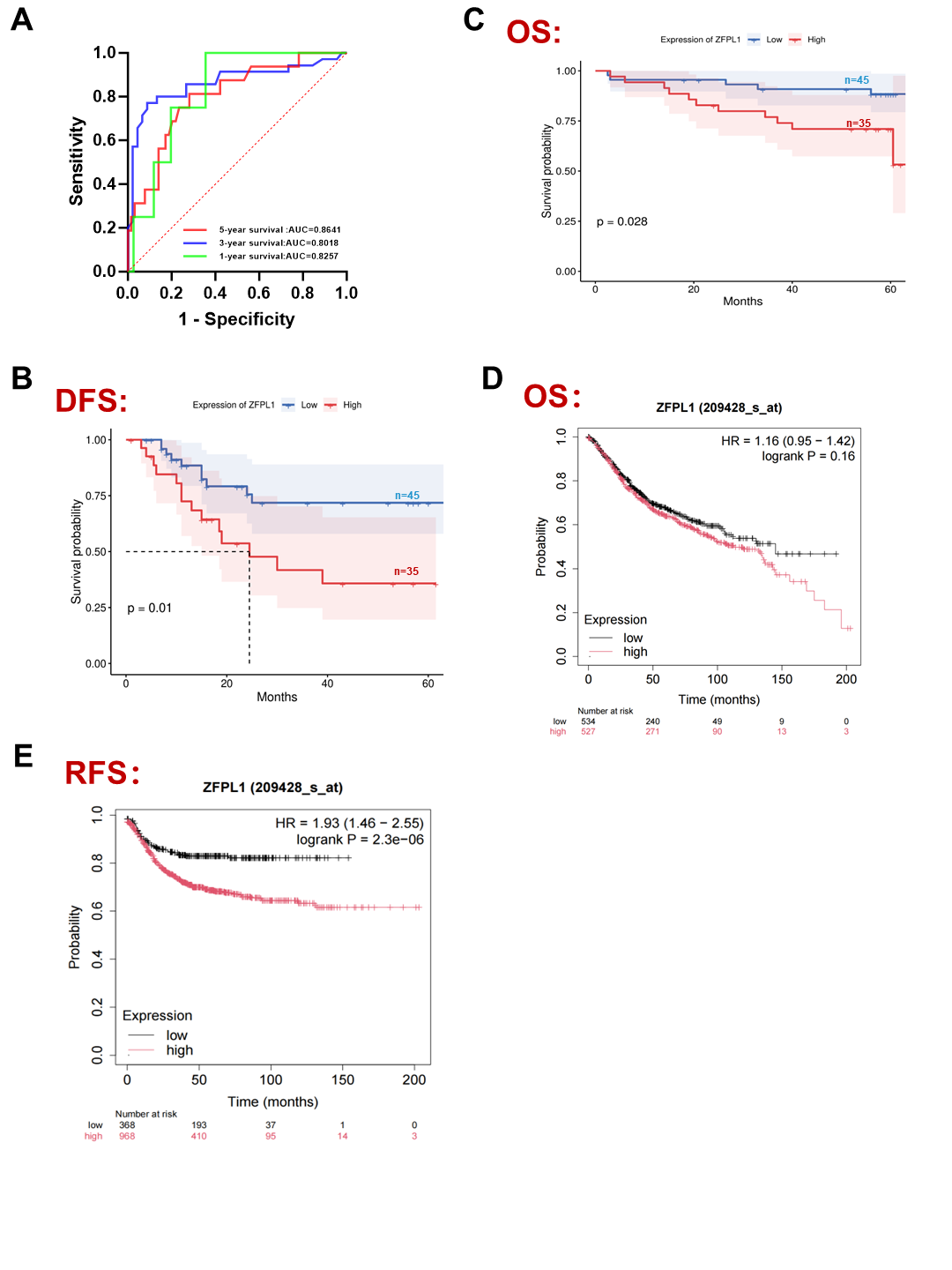


**Figure S4:**

(A-B) Verification by qRT-PCR and western blot models of ZFPL1 overexpression.

(C) CCK-8 assays were carried out to assess the effect of ZFPL1 on CRC cell proliferation. n=3 independent biological replicates.

(D) EdU assays were performed to assess the effect of ZFPL1 on CRC cell proliferation (scale bars, 50μm). n=3 independent biological replicates.

(E) Plate colony formation assay was used to evaluate the effect of ZFPL1 on CRC cell proliferation. n=3 independent biological replicates.

(F) Wound healing assays were performed to determine the migration capacities of transfected CRC cell line (scale bars, 200 μm). n=3 independent biological replicates.

(G) Transwell assays were performed to determine the invasion capacities of transfected CRC cell line (scale bars, 200 μm). n=3 independent biological replicates.

(H) Two groups of pictures showing liver metastasis from CRC.

In all statistical plots, data are expressed as the mean ± SD, one-way ANOVA (Figure S4C) and Student’s t test (Figure S4A, D, E, F, G) were used to determine statistical significance. (**P < 0.01, ***P < 0.001).


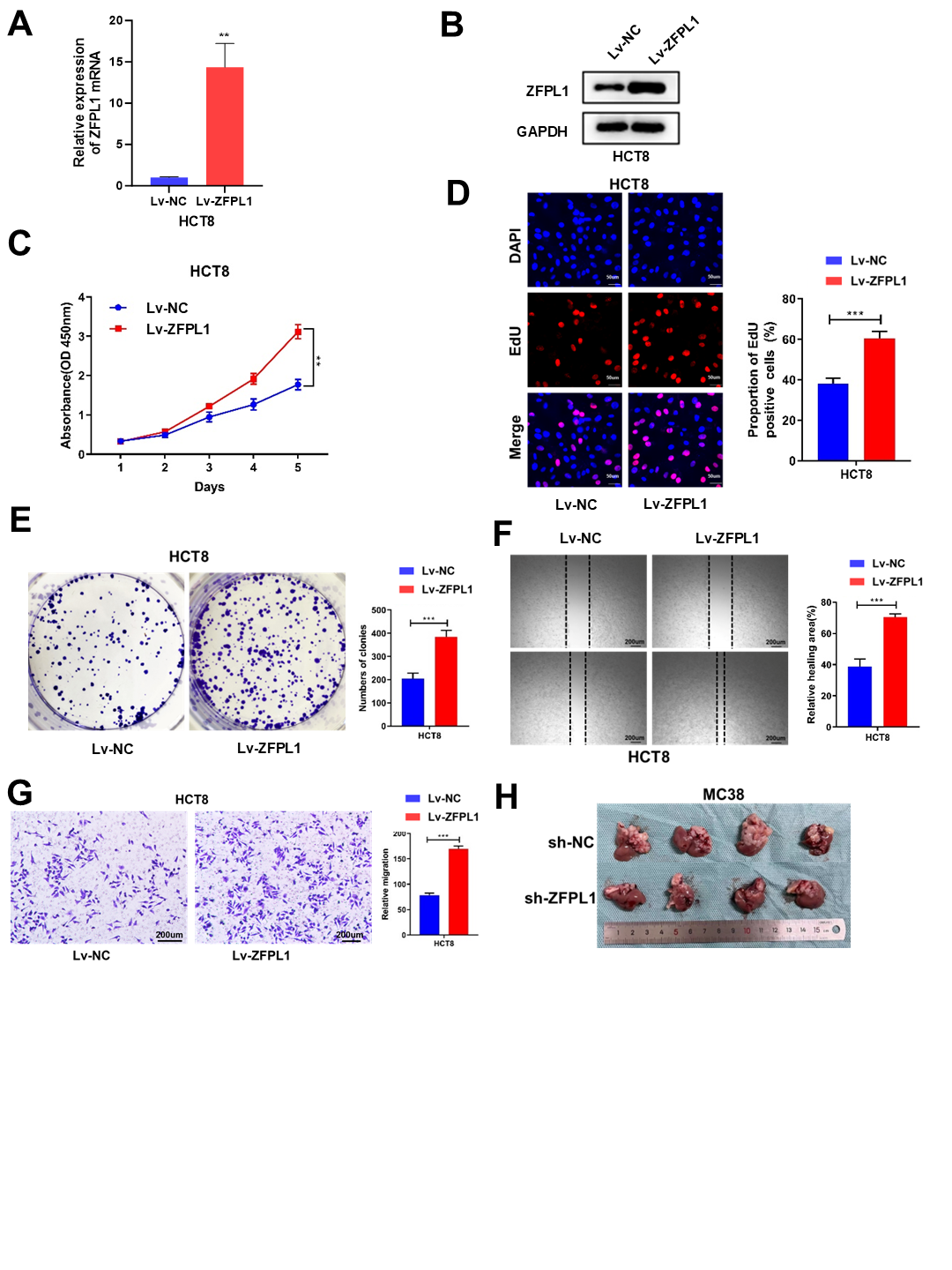


**Supplementary tables**

**Table1. The clinicopathological relevance analysis of ZFPL1 expression in 80 CRC patients**

| Characters | | Low group | High group | P value |
| --- | --- | --- | --- | --- |
|  |  | (N＝ 45) | (N ＝ 35) |  |
| Age (years) | <50 | 10 | 6 | 0.573 |
|  | ≥50 | 35 | 29 |  |
| Gender | Female | 20 | 15 | 0.887 |
|  | Male | 25 | 20 |  |
| Location | Rectum | 24 | 17 | 0.673 |
|  | Colon | 21 | 18 |  |
| Pathological type | Adenocarcinoma | 2 | 2 | 0.796 |
|  | Others | 43 | 33 |  |
| CEA (ng/ml) | < 5 | 29 | 21 | 0.684 |
|  | ≥ 5 | 16 | 14 |  |
| CA19-9 (U/ml) | ≥ 22 | 30 | 20 | 0.383 |
|  | < 22 | 15 | 15 |  |
| KRAS | Wild | 24 | 19 | 0.932 |
|  | Mutant | 21 | 16 |  |
| PNI | Negative | 39 | 29 | 0.636 |
|  | Positive | 6 | 6 |  |
| LVI | Negative | 34 | 19 | 0.046* |
|  | Positive | 11 | 16 |  |
| Diferentiation | High | 1 | 1 | 0.857 |
|  | Low+ Moderate | 44 | 34 |  |
| Lymph node metastasis | Negative | 37 | 21 | 0.027* |
|  | Positive | 8 | 14 |  |
| Tumor diameter | ≤5 cm | 24 | 8 | 0.006* |
|  | ＞5 cm | 21 | 27 |  |
| TNM stage | Ⅰ+Ⅱ | 20 | 14 | 0.690 |
|  | Ⅲ+Ⅳ | 25 | 21 |  |

Notes: PNI: Perineural Invasion; LVI: Lymph vascular Invasion.

**Table S2. Primer and sh-RNA sequences used in this study**

| Gene name | Gene symbol | Target sequence (5’-3’) |
| --- | --- | --- |
| ASS1 | Forward | TCCGTGGTTCTGGCCTACA |
|  | Reverse | GGCTTCCTCGAAGTCTTCCTT |
| ZFPL1 | Forward | GCCGATCAGTAAACACAGA |
|  | Reverse | ACTGGACGATGCACTTG |
| GAPDH | Forward | AAGCTCATTTCCTGGTATGAC |
|  | Reverse | TCTTCCTCTTGTGCTCTTGCT |
| shZFPL1#1 | shRNA | CCUAGGAAGGUGUAUGAUA |
| shZFPL1#2 | shRNA | CGACCCGCCUUGUCUGCUA |
| shZFPL1#3 | shRNA | GCUCCAAGAUAGCGACTAC |
| sh-NC | Negative control | AACTGGACTTCCAGAAGAACA |

**Table S3. Antibodies used in this study**

| Antibody | Source |
| --- | --- |
| Anti-CD8 antibody | Cell Signaling Technology |
| Anti-CD86 antibody | Cell Signaling Technology |
| Anti-CD206 antibody | Cell Signaling Technology |
| Anti- ZFPL1antibody | Abcam |
| Anti- Ki67 antibody | Abcam |
| Anti- PD1antibody | Abcam |
| Anti-GAPDH antibody | Abcam |
| Secondary antibody | Abcam |
| Anti-IgG antibody | Cell Signaling Technology |
